# Pyruvate kinase M2-mediated histone lactylation alters three-dimensional genomic architecture in polycystic ovary syndrome

**DOI:** 10.1038/s41392-025-02468-5

**Published:** 2025-11-19

**Authors:** Chuanjin Yu, Tingting Liu, Yishu Wang, Xinghui Guo, Yujie Chen, Yifan Zhao, Xia Liu, Weiwei Huang, Shuoyang Zhao, Jiaying Mo, Hongtao Hu, Pingping Lv, Xiaotao Wang, Zuwei Yang, Jiexue Pan, Guolian Ding, Jianzhong Sheng, Xinmei Liu, Hongbo Yang, He-Feng Huang

**Affiliations:** 1 Institute of Reproduction and Development, Shanghai Key Laboratory of Reproduction and Development, Obstetrics and Gynecology Hospital, Fudan University, Shanghai, China; 2 Shanghai Key Laboratory of Female Reproductive Endocrine Related Diseases, Shanghai, China; 3 Research Units of Embryo Original Diseases, Chinese Academy of Medical Sciences, Shanghai, China; 4 Zhongda Hospital, Advanced Institute for Life and Health, School of Public Health, Southeast University, Nanjing, China; 5 The International Peace Maternity and Child Health Hospital, School of Medicine, Shanghai Jiao Tong University, Shanghai, China; 6 Morphology and spatial multi-omics core facility Shanghai Institute of Nutrition and Health, Chinese Academy of Sciences, Shanghai, Chin China; 7 Assisted Reproduction Unit, Department of Obstetrics and Gynecology, Sir Run Run Shaw Hospital, Zhejiang University School of Medicine, Hangzhou, China; 8 Department of Gynecology, The First Affiliated Hospital of Nanjing Medical University, Nanjing, China; 9 Key Laboratory of Reproductive Genetics (Ministry of Education), Department of Reproductive Endocrinology, Women’s Hospital, Zhejiang University School of Medicine, Hangzhou, China

**Keywords:** Endocrine system and metabolic diseases, Epigenetics

## Abstract

Polycystic ovary syndrome (PCOS) is a frequent endocrine and metabolic imbalance that typically occurs in women of reproductive age. Its molecular pathophysiology is yet unknown, especially the ovarian cellular metabolic inefficiency that causes the transcriptional dysregulation of key genes linked to PCOS. Here, we discovered that one transcriptional-like regulator that causes PCOS is nuclear pyruvate kinase M2 (nPKM2). Using multiomics techniques, we show that enhanced lactylation of histone 3 on lysine residues 9 and 18 is linked to nPKM2 binding to the genome, changing the three-dimensional architecture of the genome. Genomic compartment switching, topologically associated domain fusion, and novel enhancer–promoter interactions subsequently enhance the expression of PCOS-related genes, including *CYP17A1* and *CYP11A1*. In mice, ectopic expression of *Pkm2* in female GCs consistently presented PCOS-like traits, such as interrupted estrous cycles, hyperandrogenism, and so on. Importantly, whole-organ tracing imaging directly unfolded the number of small follicles, which increased highly in *Pkm2*-tdtomato transgene mice compared with control. Furthermore, pharmacological inhibition of the nuclear accumulation of PKM2 mitigated PCOS-like symptoms in mice and restored a wild-type-like transcriptome. This study demonstrates the important function of PKM2-mediated histone lactylation in regulating the three-dimensional chromatin architecture and highlights PKM2 as a potential therapeutic target for PCOS treatment.

## Introduction

Polycystic ovary syndrome (PCOS) is a multifactorial and heterogeneous endocrine disease that affects an estimated 10–20% of women of reproductive age worldwide.^1–5^ It is the leading cause of miscarriage and infertility among women and is characterized by three clinical features: hyperandrogenism (clinical or biochemical), oligo-ovulation and anovulation (menstrual irregularities) and polycystic ovarian morphology (PCOM).^6^ Although investigations over several decades have revealed several possible mechanisms of PCOS,^7–10^ the etiology and pathophysiology of this disease remain elusive. Several studies have highlighted the associations between PCOS and metabolic disturbances, including obesity and insulin resistance (IR).^11,12^ The metabolic subtype represented approximately one-third of the PCOS cases. In a genome-wide association study, among the five identified susceptible sites in PCOS, the *KCNH7/FIGN* locus (chr2 q24.2–q24.3) was found to be linked to the metabolic subtype.^13^ In a phenome-wide association study, Joo and colleagues reported a correlation between the PCOS polygenic risk score and obesity and obesity-related phenotypes.^14^ In addition to symptoms of metabolic disorders, patients with PCOS have abnormal metabolite levels in the ovarian microenvironment. Patients with PCOS have higher levels of insulin in the follicular fluid (FF) and exhibit abnormal glycolysis in granulosa cells (GCs),^7,15^ which suggests that cellular metabolism in the ovary is different from that in the control group.

Epigenetics plays a critical role in regulating transcription by integrating external environmental cues. Accumulating evidence indicates interactions between metabolism and epigenetics.^16,17^ Metabolites can serve as substrates for epigenetic modifications of histones, DNA, or chromatin regulators and can thereby regulate gene expression.^18–20^ For example, α-ketoglutarate (α-KG) is essential for DNA and histone demethylation, whereas acetyl-CoA is required for histone acetylation.^19,20^ Supplementing with S-adenosylmethionine (SAM) partially restores PCOS traits by providing the methyl groups needed for epigenetic regulation.^9,21^ PCOS GCs exhibit a shift in histone modifier expression, characterized by the upregulation of the methyltransferases SUV39H1 (H3K9me3) and EZH2 (H3K27me3) concurrent with the downregulation of the erasing enzymes HDAC3 (deacetylase) and KDM1A (demethylase).^22^ These studies demonstrate the significance of metabolism and epigenetics in PCOS. However, the detailed mechanism by which metabolic disorders drive changes in the epigenomic landscape, leading to the dysregulation of PCOS-related genes, remains an area of active investigation.

Glycolysis is the primary metabolic pathway through which GCs provide energy for follicular development.^23,24^ Pyruvate kinase (PK) is an enzyme that plays a key role in the rate-limiting process of glycolysis. It catalyzes the transfer of the phosphate group from phosphoenolpyruvate to ADP, thereby generating ATP and pyruvate. Pyruvate is subsequently converted into lactate, the final product of glycolysis. The regulation of gene expression via lactate-induced histone lactylation is a critical mechanism for maintaining normal physiological function.^25^ Recently, the nuclear lactate concentration in mouse 2-cell-stage embryos was shown to be notably greater than that in the cytosol and other embryonic developmental stages.^26^ The decrease in histone H3K18 lactylation (H3K18la) prevented major zygotic genome activation (ZGA) and led to the blockade of embryonic development at the 2-cell stage. H3K18la was also found to be able to increase the efficiency of induced pluripotent stem cell reprogramming through metabolic switching and p300 recruitment.^27,28^ Dysregulated lactate homeostasis is a key factor in the pathogenesis of numerous diseases.^29,30^ For example, overactivation of glycolysis and lactate accumulation in microglia in patients with Alzheimer’s disease disrupt microglial homeostasis and promote neuroinflammation via histone H4 lysine 12 lactylation.^31^ Zhang et al. reported that a deficiency in lactate efflux is associated with H3K18la upregulation and the progression of atherosclerosis.^32^

We have previously shown that the GCs microenvironment is disrupted by dysregulated expression of *PGK1*, a key glycolytic gene, which contributes to PCOS development.^15^ To further dissect the relationship between the dysregulation of glycolysis and PCOS pathogenesis, in this study, Mendelian randomization (MR) analysis was used to investigate the causal associations between genetically predicted differences in glycolysis enzymes and the risk of PCOS and demonstrated that PKM2 might contribute to the genetic determinants of PCOS. Furthermore, we profiled the differential proteome of GCs between control individuals and patients with PCOS and detected elevated levels of PKM2, a key glycolytic enzyme, in the GCs of patients with PCOS. This upregulation was accompanied by the nuclear accumulation of PKM2 in the GCs of patients. Next, we confirmed that upregulation of PKM2 is critical for PCOS pathogenesis because its ectopic expression in GCs recapitulates the PCOS phenotype in mice. Furthermore, we investigated the important function of PKM2 in the nucleus by constructing a cell line in which PKM2 was localized to the nucleus. We profiled the genome-wide PKM2 binding and histone lactylation landscape in this cell line and identified the colocalization of PKM2 with H3K9la and H3K18la histone modifications. This PKM2-dependent histone modification altered the three-dimensional (3D) genome architecture and promoted the expression of androgen synthesis genes, including *CYP17A1* and *CYP11A1*, as well as the PCOS-related genes *WNT4* and *PADI3*, by forming new enhancer‒promoter loops. Importantly, the small molecule TEPP46, which blocks the nuclear accumulation of PKM2 and thus disrupts this metabolic–epigenetic axis, inhibits PCOS pathogenesis in mice. Overall, our study establishes the critical function of nuclear PKM2 as a transcriptional regulator by affecting the histone lactylome and highlights the distinguished function of histone lactylation in modulating the 3D genome conformation. These findings provide novel avenues for PCOS therapy.

## Results

### Combined proteomics and Mendelian randomization analysis of the correlation between glycolytic enzymes and PCOS

**Fig. 1:**
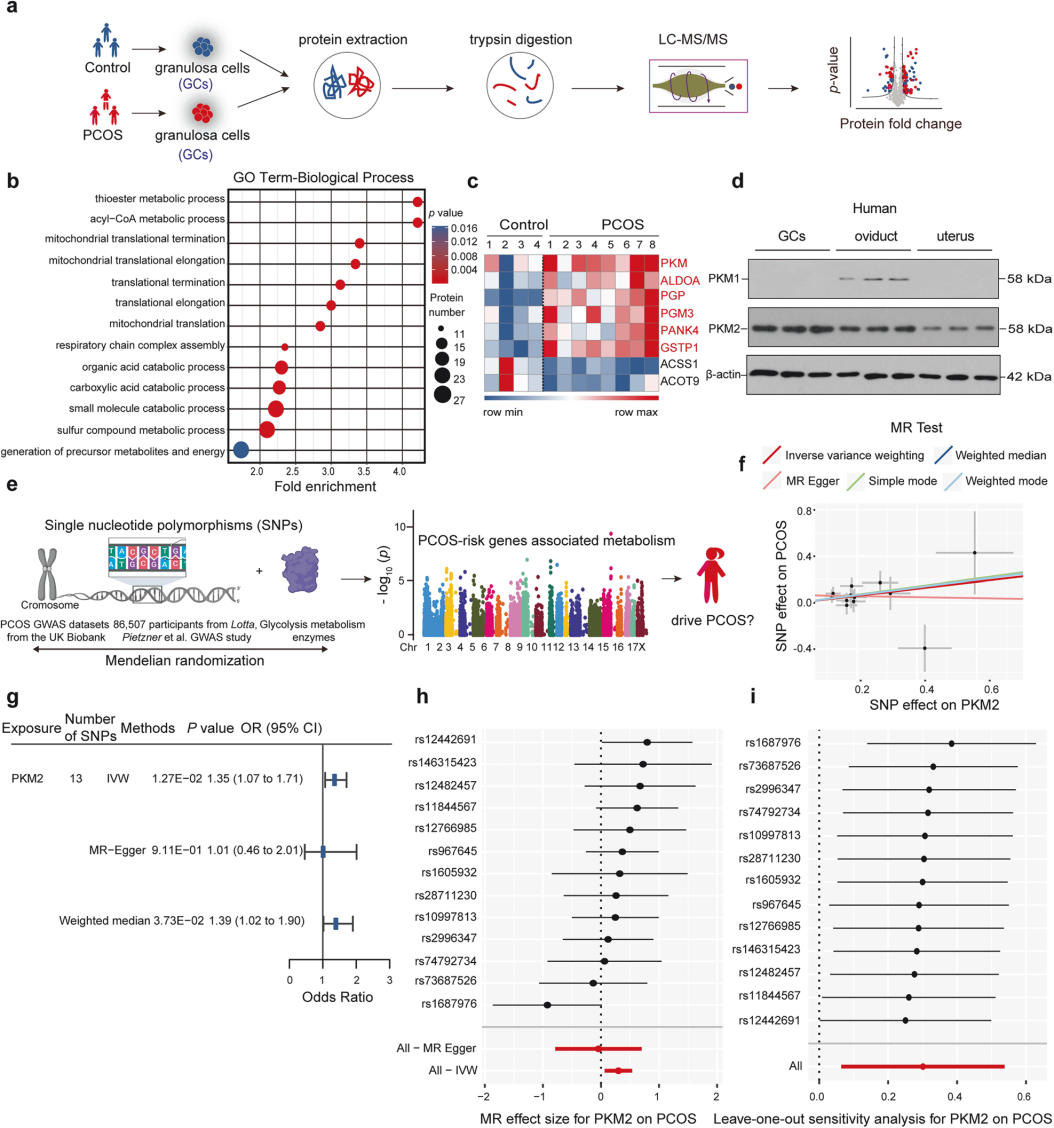
Proteomic analysis of luteinized granulosa cells (GCs) in PCOS, and the association of PKM2 with PCOS was analyzed via the MR approach. **a** The proteomics workflow included protein extraction from luteinized GCs. **b** Dot plot of gene enrichment pathways for proteins enriched in patients with PCOS. **c** Heatmap of metabolic enzymes related to glycolysis or other metabolic processes (upregulated proteins shown in red) in luteinized GCs from control (*n* = 4) and PCOS (*n* = 8) patients. Peptide levels were determined via proteomics analysis. *p* < 0.05 and fold change *≥* 1.3. **d** Western blot showing the PKM1 and PKM2 expression patterns in human reproductive-related cells or organs (*n* = 3). **e** Flow chart of the two-sample Mendelian randomization analysis using GWAS genetic data to investigate the causal relationship between glycolytic enzymes and PCOS (partially created with BioRender.com). **f** Scatter plots showing estimates of the risk of PCOS according to significant metabolic enzyme exposure during glycolysis. **g** Forest plot demonstrating significant MR results for key enzymes involved in glycolysis that may impact the risk of PCOS. The effect estimates represent the ORs for PCOS per 1-SD increment in PKM2. The error bars represent 95% CIs. **h** Forest plots illustrating the genetically predicted MR effect sizes of individual and combined SNPs for each exposure–outcome pair. **i** IVW MR regression results for the leave one SNP out analysis in the sensitivity analysis of PKM2. PKM2 pyruvate kinase isozyme type M2, IV inverse variance, IVW inverse variance weighted, MR Mendelian randomization, SNP single-nucleotide polymorphism

Glycolysis serves as the major energy source for follicular growth and development, particularly in GCs, which heavily depend on this pathway to support follicle progression from the secondary to preovulatory stages.^33,34^ Research studies have demonstrated that patients with PCOS frequently exhibit abnormal glucose metabolism, with impaired glycolysis in granulosa cells (GCs) being one of the reasons for follicle maturation and oocyte development failure in PCOS.^15,35^ To investigate the link between PCOS and metabolic enzyme dysfunction, we collected GCs from patients undergoing in vitro fertilization (IVF), including eight individuals with PCOS and four controls with tubular infertility. We analyzed the samples via high-resolution liquid chromatography‒tandem mass spectrometry (LC‒MS/MS) (Fig. 1a) and identified 430 differentially expressed proteins between the PCOS patients and the controls (Supplementary Table 1). The metabolic process was linked to 7 of the 13 highest-ranked enriched pathways during gene ontology (GO) analysis of the differentially expressed proteins (Fig. 1b), indicating the involvement of metabolic dysfunction in PCOS pathogenesis. The heatmap data revealed that some metabolic enzymes, including PKM, one of the key rate-limiting glycolytic enzymes, were upregulated (Fig. 1c) and were our interest. The PKM locus transcribes two isoforms, PKM1 and PKM2, depending on the alternative splicing of exons 9 and 10 (Supplementary Fig. 1a). Intriguingly, PKM2 was found to be predominantly expressed in female reproductive-associated organs, particularly in GCs, and this pattern was consistent across humans (Fig. 1d) and mice (Supplementary Fig. 1b, c). Proteomic data from granulosa cells indicated that PKM2 was closely associated with PCOS. Moreover, to investigate the causal associations between genetic differences in multiple key enzymes involved in glycolysis and the risk of PCOS, Mendelian randomization (MR) analysis was conducted (Fig. 1e). Elevated PKM2 levels, coupled with reduced circulating TPI1 and FBP1 levels (Supplementary Fig. 1d-f), demonstrated a causal association with PCOS risk, with PKM2 emerging as the primary driver among glycolysis-related factors in multivariable MR analyses (Supplementary Fig. 1g). The scatter plot revealed that the effect sizes of SNP-PKM2 and SNP-PCOS were positively associated (Fig. 1f). Moreover, the results from the IVW method suggest a positive correlation between PKM2 and a greater genetic predisposition to PCOS (odds ratio [OR] 1.35, 95% confidence interval [CI] 1.07–1.71). The consistency of the results was confirmed by both the weighted median (OR 1.39, 95% CI 1.02–1.90) and the MR‒Egger (OR 1.01, 95% CI 0.46–2.01) methods (Fig. 1g). In the sensitivity analysis, MR‒Egger regression did not indicate significant directional pleiotropy (MR‒Egger intercept 0.03, SE 0.16, *p* = 0.87). There were no outliers detected by the MR pleiotropy residual sum and outlier (MR-PRESSO) test (global test *p* = 0.53). Furthermore, no evidence of heterogeneity among the IVs for PKM2 was observed by the MR‒Egger and IVW methods used in Cochran’s *Q* test (p_MR-Egger_ = 0.76, p_IVW_ = 0.93) (Supplementary Table 2). Forest plots showed the MR effect sizes of individual and combined SNPs for each exposure‒outcome pair and indicated that PKM2 may contribute to genetic determinants of PCOS (Fig. 1h), and the leave‒one-out method revealed that the causal inference was not substantially influenced by any individual SNP (Fig. 1i). Taken together, combined proteomics and Mendelian randomization analysis data suggest that PKM2 might be most likely associated with PCOS.

### Upregulation and nuclear accumulation of PKM2 in the GCs of patients with PCOS

**Fig. 2:**
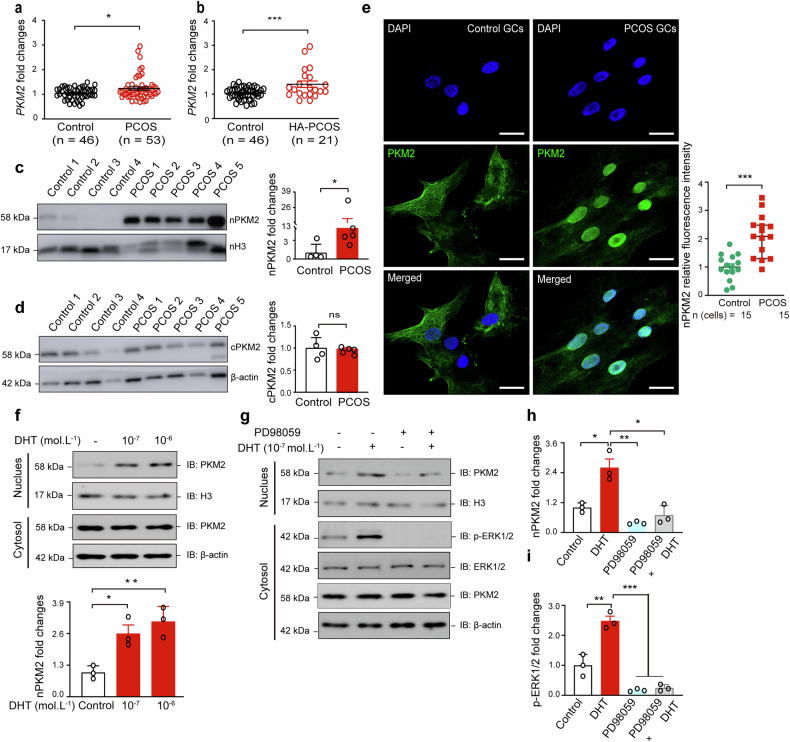
PKM2 accumulation in the nucleus of GCs from patients with PCOS. **a** Relative mRNA expression of *PKM2* in human luteinized GCs in the control and PCOS groups. *n* = 46 in the control group and *n* = 53 in the PCOS group. The data are presented as the means ± SEMs. The *p* values were determined by a two-tailed unpaired Student’s *t* test. * *p* < 0.05, *** *p* < 0.001. **b** Relative mRNA expression of *PKM2* in human luteinized GCs in the control and high androgen PCOS (HA-PCOS) groups. *n* = 46 in the control group and *n* = 21 in the PCOS group. The data are presented as the means ± SEMs. The *p* values were determined by a two-tailed unpaired Student’s *t* test. *** *p* < 0.001. **c**, **d** Nuclear PKM2 and cytoplasmic PKM2 levels were determined via western blotting of protein samples from luteinized GCs in the control and PCOS groups. *n* = 4 in the control group and *n* = 5 in the PCOS group. The data are presented as the means ± SEMs. Statistical tests were performed via the Mann‒Whitney test, * *p* < 0.05. **e** Immunofluorescence data showing PKM2 expr**e**ssion profiles in the nuclei of control and PCOS GCs. Scale bar = 20 µm. *n* = 15 cells in each group. The data are presented as the means ± SEMs. The *p* values were determined by a two-tailed un*p*aired Student’s *t* test. *** *p* < 0.001. **f** Western blot showing PKM2 levels in the cytosolic and nuclear lysates of KGN cells treated with dihydrotestosterone (DHT; 10^–7^ or 10^–6^ mol/L) for 24 h. Data are presented as the means ± SEMs. The *p* values were determined via two-tailed unpaired Student’s *t* tests. * *p* < 0.05, ** *p* < 0.01. **g**–**i** Western blot showing PKM2 levels in the cytosolic and nuclear lysates of KGN cells treated with DHT (10^–7^ mol/L) or PD98059 (20 µM) for 24 h. Data are presented as the means ± SEMs. The *p* values were determined by a two-tailed unpaired Student’s *t* test. * *p* < 0.05, ** *p* < 0.01, *** *p* < 0.001. The experiments were performed three times (**c**–**g**)

To further prove that PKM2 plays an important role in the pathogenesis of PCOS, we collected more GCs from patients to confirm its function. The qPCR data for GCs from patients revealed that *PKM2* levels were greater in PCOS GCs, especially in high androgen PCOS (HA-PCOS) GCs, than in control GCs (Fig. 2a, b), and RNA-seq also revealed that *PKM2* levels were greater in PCOS GCs (Supplementary Fig. 2a). Additionally, *PKM2* expression was positively correlated with testosterone (TT) levels and the ratio of luteinizing and follicle-stimulating hormones (LH/FSH), two clinical features of PCOS (Supplementary Fig. 2b, c). These findings demonstrate a close association between PKM2 upregulation and PCOS pathogenesis.

Although glycolysis typically occurs in the cytoplasm, PKM2 also functions in the nucleus in distinct ways. Previous cancer studies have shown that upregulated glycolysis leads to the nuclear accumulation of PKM2, which can influence histone modification.^36^ We investigated the distribution of PKM2 in the nucleus and cytoplasm of PCOS and control GC cells and detected similar levels of cytoplasmic PKM2 in these cells; however, clinical PCOS GCs presented significantly increased nuclear PKM2 levels, as evidenced by Western blot and immunostaining analyses (Fig. 2c–e). Given that hyperandrogenism is one of the main characteristics of PCOS, we hypothesized that high androgen levels are involved in the nuclear translocation of PKM2. To test this hypothesis, we treated KGN (a human ovarian granulosa-like cell line) with dihydrotestosterone (DHT) and observed a significant increase in the nuclear accumulation of PKM2 (Fig. 2f). In a previous study, ERK was shown to phosphorylate PKM2 at Ser 37, facilitating its binding to importin α5 and subsequent translocation into the nucleus.^37^ We found that PD98059, an ERK1/2-specific inhibitor, suppressed the nuclear localization of PKM2 (Fig. 2g–i). Collectively, these findings demonstrate that nuclear PKM2 levels are significantly increased in PCOS GCs and suggest that PKM2 translocates to the nucleus through the ERK1/2 signaling pathway.

### Ectopic expression of PKM2 in GCs induces the PCOS phenotype in mice

**Fig. 3:**
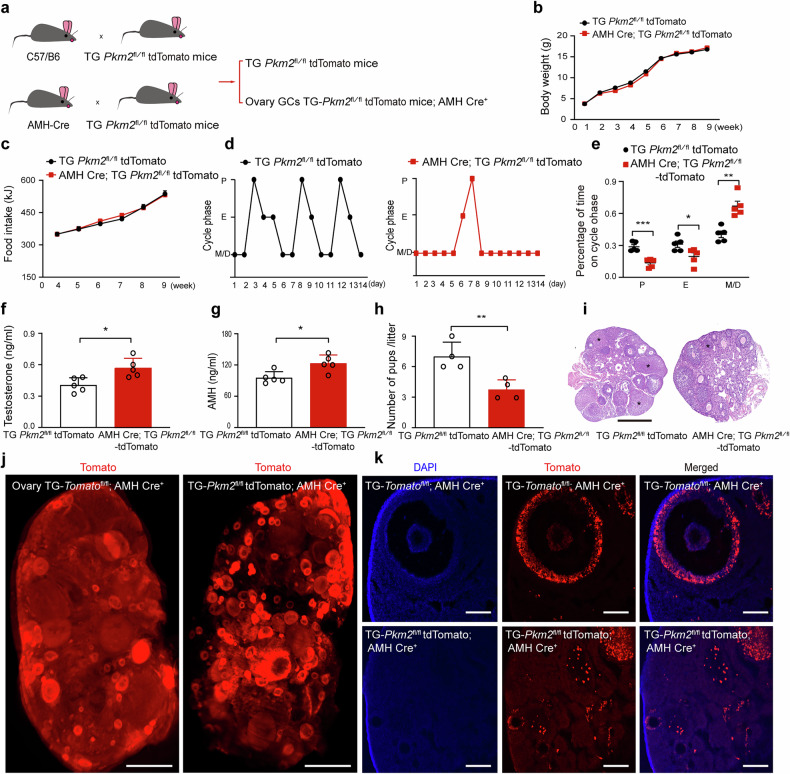
Granulosa cell *Pkm2* promotes PCOS-like reproductive dysfunction. **a** Schematic of the genetic cross between the R26-LSL-Tomato or R26-LSL-*Pkm2*-tdTomato mouse line and the AMH-Cre line. **b** Body mass of TG *Pkm2*^fl^/^fl^ tdTomato and TG- *Pkm2*^fl^/^fl^ tdTomato;AMH Cre^+^ mice fed a normal diet for 9 weeks (*n* = 3 per group). **c** Cumulative food intake of TG *Pkm*2^fl^/^fl^ tdTomato mice and TG- *Pkm*2^fl^/^fl^ tdTomato;AMH Cre^+^ mice fed a normal diet over a period of 9 weeks (*n* = 3 per group). **d**, **e** Continuous monitoring of the estrus stage in the indicated groups. *n* = 5 mice in each group. The data are presented as the means ± SEMs. The *p* values were determined by a two-tailed unpaired Student’s *t* test. * *p* < 0.05, ** *p* < 0.01, *** *p* < 0.001. **f**, **g** Testosterone and anti-Mullerian hormone (AMH) levels were detected by enzyme-linked immunosorbent assay (ELISA) in the indicated groups. *n* = 5 mice in each group. The data are presented as the means ± SEMs. The *p* values were determined via two-tailed unpaired Student’s *t* tests. * *p* < 0.05. **h** The number of pups in the treated groups. *n* = 5 mice in each group. The data are prese*n*ted as the means ± SEMs. The *p* values were determined by a two-tailed un*p*aired Student’s *t* test. ** *p* < 0.01. **i** Hematoxylin‒eosin staining shows images of representat**i**ve treated mouse ovaries. * indicates the corpus luteum. Scale bar = 500 µm. *n* = 3 mice in each group. **j** Images of whole-mount ovary sections from TG- *Pkm2*^fl^/^fl^ tdTomato;AMH Cre^+^ and TG-Tomato^fl^/^fl^;AMH Cre^+^ mice. Scale bars = 100 µm^.^ *n* = 3 mice in each group. **k** Immunostained images showing tdTomato expression in ovary sections. Scale bars = 100 µm. The data are presented as the means ± SDs. *n* = 3 mice in each group. The experiments were performed three times (**b**–**k**)

To investigate whether PKM2 upregulation is crucial for PCOS development, we generated a mouse line that specifically ectopically expressed *Pkm2* in ovarian GCs by crossing Rosa26-loxP-Stop-loxP-*Pkm2*-tdTomato (R26-LSL-*Pkm2*-tdTomato) knock-in mice with AMH-Cre knock-in mice (Fig. 3a). After nine weeks on a normal diet, TG (*Pkm2*-tdTomato^fl^/^fl^) (hereinafter referred to as the control) mice and AMH Cre; TG (*Pkm2*-tdTomato^fl^/^fl^) (hereinafter referred to as *Pkm2*-OE) mice presented similar weight gain and food intake (Fig. 3b, c). We also found that, compared with those in the control, *Pkm2* expression levels were high in the ovaries but not in other tissues of *Pkm2*-OE mice (Supplementary Fig. 2d). We then assessed the main criteria for PCOS diagnosis, namely, hyperandrogenism and oligoanovulation, in the transgenic lines. Compared with control *Pkm2*-OE mice, female Pkm2-OE mice consistently presented PCOS-like traits, such as interrupted estrous cycles, which involved a longer period of time spent in metestrus and diestrus (Fig. 3d, e). From postnatal week 9, female *Pkm2*-OE mice had significantly higher TT and anti-Mullerian hormone (AMH) levels and a lower birth rate than did the control group (Fig. 3f–h). In addition, hematoxylin and eosin (H&E) staining revealed that the number of corpora lutea decreased, and the number of small follicles increased notably in *Pkm2*-OE mice compared with those in control mice (Fig. 3i). Importantly, whole-mount fluorescence and immunostaining revealed that tdTomato expression was predominantly localized in the ovarian GCs, and *Pkm2*-OE ovaries contained many small follicles, in contrast to the fewer, larger follicles in the control group, indicating oligo-ovulation in *Pkm2*-OE mice (Fig. 3j, k).

To further confirm whether *Pkm2* ectopic expression in GCs significantly contributes to PCOS pathogenesis, we injected an adeno-associated virus carrying *Pkm2* into the ovaries of 6-week-old mice. Ectopic expression of *Pkm2* caused a cascade of abnormalities, including aberrant estrus cycles, antral follicle defects, increased fasting blood glucose, and impaired glucose and insulin tolerance (Supplementary Fig. 3). These findings confirm that the ectopic expression of *Pkm2* in GCs is sufficient to induce a PCOS-like phenotype in mice.

### Nuclear accumulation of PKM2 changes the global histone lactylation landscape and enhances the expression of PCOS-related genes

Lactate regulates histone lactylation.^25,31,38^ To explore whether *PKM2* upregulation causes epigenomic fluctuations and affects the expression of PCOS-related genes, we generated several transgenic KGN lines. First, we confirmed that ectopic expression of *PKM2* in cells not only resulted in metabolic disturbance phenotypes, such as increased glucose consumption, a higher extracellular acidification rate (ECAR), and a lower oxygen consumption rate (OCR) (Supplementary Fig. 4a–c) but also led to increased lactate levels (Supplementary Fig. 4d). Conversely, knocking out *PKM2* produced the opposite effects (Supplementary Fig. 4e, f). The metabolome data also revealed that PKM2 was closely associated with the glycolysis pathway lactate (Supplementary Fig. 5).

**Fig. 4:**
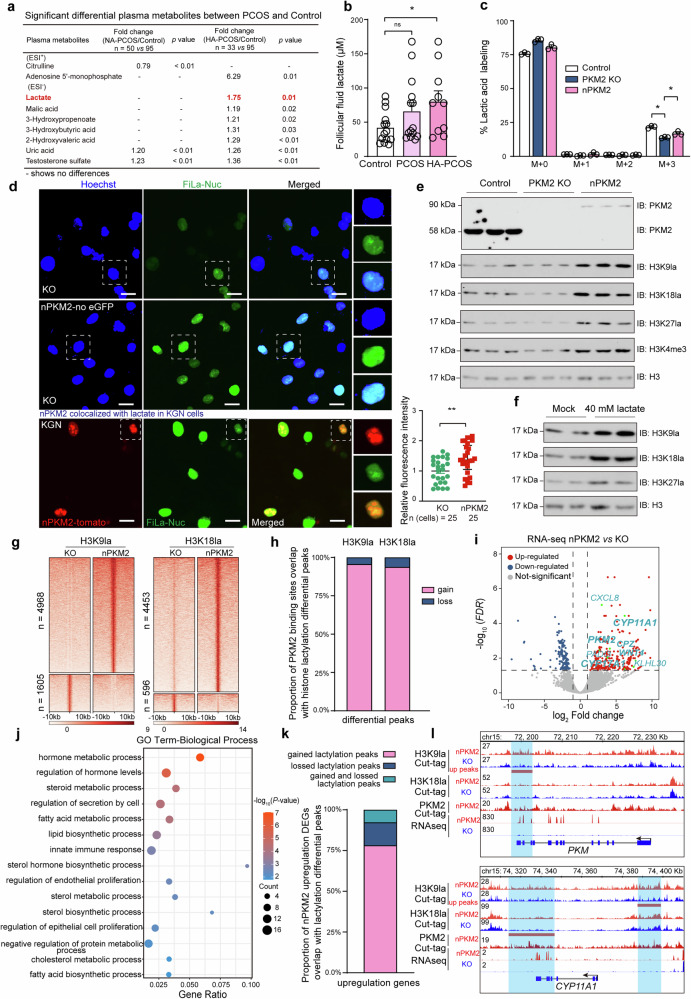
Nuclear PKM2-induced global histone lactylation and upregulation of PCOS-related genes. **a** Differential plasma metabolites were identified among the Non-high androgen PCOS (NA-PCOS), High-androgen PCOS (HA-PCOS), and control groups via UHPLC‒MS. “-” indicates no significant difference. *n* = 95 in the control group, *n* = 50 in the NA-PCOS group and *n* = 33 in the HA-PCOS group. **b** The lactate level in the follicular fluid of the control, NA-PCOS and HA-PCOS patients was determined via a lactate probe*. n* = 14 in the control group, *n* = 14 in the NA-PCOS group and *n* = 10 in the HA-PCOS group. The data are presented as the means ± SEMs. The *p* values were determined by a two-tailed unpaired Student’s *t* test. ns indicates no significant difference, * *p* < 0.05. **c** Labeling pattern of lactate from ^13^C-glucose in PKM2 knockout (KO) and nPKM2 cells (generated by introducing nuclear PKM2 into the KO cells). M + 0 indicates the fraction of the compound with an unlabeled molecular mass, M + 1 indicates the fraction of the compound with 1 heavy label (one ^13^C-labeled carbon), and so on. *n* = 3 in each group. The data are presented as the means ± SEMs. The *p* values were determined by a two-tailed unpaired Student’s *t* test. * *p* < 0.05. **d** Fluorescence images of lactate in the nucleus were acquired from KO and nPKM2 cells via the nuclear lactate sensor FiLa-Nuc, and the overexpressed nuclear PKM2 colocalized with the lactate sensor FiLa-Nuc in wild-type KGN cells. The lactate sensor FiLa-Nuc and nuclear PKM2 merged reporter gene tomato (nPKM2-tomato) were expressed in cells via the lentivirus system. *n* = 25 cells in each group. The data are presented as the means ± SEMs. The *p* values were determined by a two-tailed unpaired Student’s *t* test. ** *p* < 0.01. The experiments were performed three times. **e** Western blot showing H3K9la, H3K18la, H3K27la, and H3K4me3 in nPKM2 and PKM2 KO cells. *n* = 3 in each group. **f** Western blot showing histone lactylation of H3K9, H3K18, and H3K27 in KGN cells after treatment with 40 mM lactic acid for 24 h. *n* = 2 in each group. **g** Heatmap illustrating the differential peak (gain and loss) signal distributions for H3K9la and H3K18la in KO and nPKM2 cells. The histone lactylation levels were more enriched in nPKM2 cells (4968 gained vs. 1605 lost peaks in H3K9la and 4453 gained vs. 596 lost peaks in H3K18la). *n* = 2 in each group. **h** Proportio*n* of H3K9la and H3K18la differential peaks overlapping with the PKM2 binding sites. *n* = 2 in each group. **i** Volcano plot showing the differentially expressed genes ( | fold change |>= 2, *p* < 0.05) between KO and nPKM2 cells; the u*p*regulated genes associated with PCOS are highly labeled. *n* = 2 in each group. **j** Gene Ontology (GO) biological process analysis of nPKM2/KO RNA-seq-upregulated genes. *n* = 2 in each group. **k** Proportion of upregulated genes overlapping with differential peaks associated with histone lactylation (H3K9la and H3K18la). *n* = 2 in each group. **l** Integrative genomics viewer snapshot showing histone lactylation, nPKM2 CUT&TAG, and RNAseq signal distribution at the *PKM2* and *CYP11A1* loci. The blue bars indicate the differential histone lactylation binding sites. The vertical scale is shown on the left side of the box. *n* = 2 in each group. The experiments were performed three times (**c**–**e**)

Notably, the patient plasma metabolome data revealed that the lactate concentration was 1.75 times greater in the high-androgen PCOS (HA-PCOS) group than in the control group; however, no differences were detected between the non-high-androgen PCOS (NA-PCOS) and control groups (Fig. 4a). As detected by a sensitive lactate probe,^39^ we found that the lactate levels in the follicular fluid of HA-PCOS patients were significantly greater than those in the controls and that there were no differences between the NA-PCOS patients and the controls (Fig. 4b), which further indicated that the association of androgen and lactate may play a vital role in the ovarian microenvironment. Before examining how elevated lactate levels affect the epigenome of GCs, we simplified our study system. As mentioned above, the amount of nuclear PKM2 was elevated in the GCs of patients with PCOS. To eliminate the interference of cytoplasmic PKM2 and investigate whether nuclear PKM2 could regulate gene expression independent of its cytoplasmic activity, we utilized the CRISPR/Cas9 system to generate *PKM2*-knockout KGN single clones (Supplementary Fig. 6a–e) and then constructed a nuclear *PKM2*-overexpressing cell line by fusing *PKM2* with nuclear localization signal sequences at both the N- and C-termini (Supplementary Fig. 6f) in the parental single clones (hereinafter referred to as nPKM2). We first checked the lactate levels via a ^13^C-glucose tracking assay, in which the cells were cultured in medium containing ^13^C-glucose, which ultimately turned into ^13^C-labeled lactate. Lactate levels were significantly decreased in *PKM2*-KO cells and markedly increased when nuclear *PKM2* was replenished (Fig. 4c). To further confirm whether the lactate level in live cells is consistent with the ^13^C-labeled data, the nuclear lactate sensor FiLa-Nuc system^39^ was used to overexpress the nuclear *PKM2*-merged reporter gene *tomato* (nPKM2-tomato), and letivirus vectors were constructed. The results revealed a significantly larger pool of lactate in the nucleus of nPKM2 cells than in that of KO cells, and nPKM2-tomato colocalized with lactate in a certain area of the nucleus (Fig. 4d), which further indicated the possibility of nuclear PKM2-derived histone lactylation. To determine which histone modifications were significantly affected by the change in *PKM2*, western blot analysis of H3K9la, H3K18la, and H3K27la revealed significant increases in H3K9la, H3K18la, and H3K27la in nPKM2 cells (Fig. 4e). When we supplemented cultured nonengineered KGN cells with 40 mM lactate in the medium, the additional lactate also induced significant lactylation at the H3K9, H3K18, and H3K27 sites (Fig. 4f), confirming that these three histone sites are sensitive to changes in glycolysis and lactate levels.

To profile the epigenomic shift upon nuclear accumulation of *PKM2*, we performed CUT&Tag for an array of histone markers, including H3K9la, H3K18la, H3K27la, H3K4me3, H3K27ac, and H3K27me3, in nPKM2 and parental PKM2-KO cells, with two replicates for each sample (Supplementary Fig. 7a). Thousands of differential peaks were identified for each mark. More than 70% of the differential peaks of H3K9la, H3K18la, and H3K27la were detected in nPKM2 cells (Fig. 4g and Supplementary Fig. 7b), which is consistent with the elevated lactate levels in nPKM2 cells.

PKM2 can interact with the epigenetic modulators P300, HIF1α and STAT3.^31,40,41^ To explore the potential binding sites of PKM2 in the genome, we performed PKM2 CUT&Tag in nPKM2 cells and identified 7714 binding sites (Supplementary Fig. 7c). GO term analysis demonstrated that PKM2 binding-associated genes were abundant in glycolytic and pyruvate metabolic processes (Supplementary Fig. 7d), implying that PKM2 has a direct role in regulating the expression of genes associated with glycolysis. In addition, PKM2 binding sites overlapped with active marks, such as H3K9la, H3K18la, H3K4me3, and H3K27ac, but excluded the repressive mark H3K27me3 (Supplementary Fig. 7c and 7e). We further investigated the association between PKM2 binding and dynamic changes in histone modifications and found that the majority of the overlapping differential peaks of H3K9la and H3K18la with PKM2 binding sites were the gained peaks in nPKM2 cells (Fig. 4h).

Next, we investigated the mechanisms by which epigenetic alterations regulate transcription. We identified 266 upregulated and 154 downregulated genes in nPKM2 cells compared with PKM2-KO cells (Fig. 4i and Supplementary Table 3). The upregulated genes were most enriched in hormone metabolic processes and cholesterol biosynthetic processes, and the expression of several critical PCOS genes, such as *CYP17A1*, *CYP11A1*, *CPZ* and *PADI3*, was dramatically increased in nPKM2 cells (Fig. 4i, j and Supplementary Fig. 8a), indicating that nuclear PKM2 can induce PCOS-like transcriptome shifts in cells. The cell cycle pathway was the primary pathway enriched with the downregulated genes (Supplementary Fig. 8b and 8c). To determine which histone modification contributes the most to gene activation, we plotted the different histone mark signals at the transcription start sites (TSSs) of 266 upregulated genes and found that histone lactylation signals, together with H3K4me3, were significantly enriched in nPKM2 cells (Supplementary Fig. 8d). We further correlated the upregulated genes with the merged H3K9la, H3K18la, and H3K27la differential peaks and found that more than 75% of the associated peaks were enriched in nPKM2 cells. GO analysis revealed an enrichment of glycolytic and pyruvate metabolic processes (Fig. 4k and Supplementary Fig. 8e), which highlights the active role of histone lactylation in gene regulation. We selected two gene loci, *PKM2* and *CYP11A1*, for detailed inspection to confirm our results. The binding of PKM2 at both loci indicates positive feedback regulation of PCOS-related genes, with both H3K9la and H3K18la signals increasing with increasing gene expression (Fig. 4l). Using de novo motif analysis of the 4968 H3K9la peaks in nPKM2 cells, we found that several transcription factors (TFs), including HNF4G and FOS, likely contribute to transcriptional regulation (Supplementary Fig. 8f). Notably, the androgen receptor (AR), which plays a significant role in PCOS pathogenesis, was at the top two of the de novo TF motif list (Supplementary Fig. 8 f).

Overall, our data revealed that the nuclear localization of PKM2 and increased lactate levels are associated with increased histone lactylation and increased expression of PCOS-related genes.

### Increased histone lactylation alters the 3D organization of the genome

**Fig. 5:**
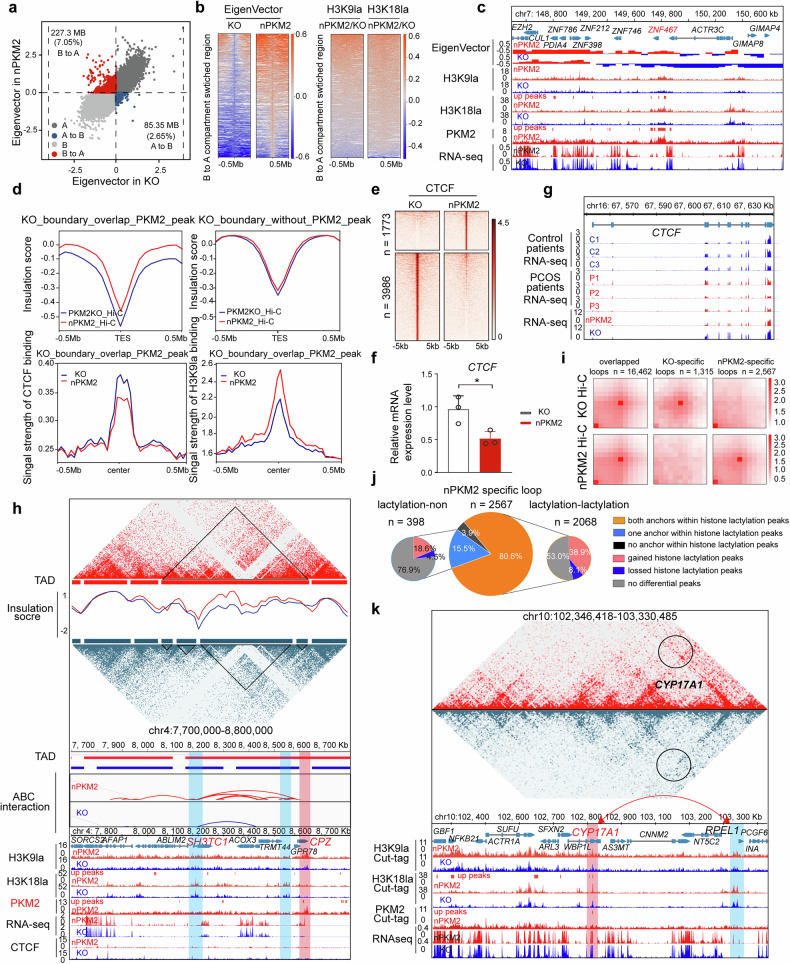
Nuclear PKM2-induced histone lactylation drives changes in three-dimensional genome organization in KGN cells. **a** Genomic analysis revealed that nPKM2 cells harbored more active chromatin (compartment A), with a total size of 1.19 Gbp, than 1.15 Gbp in KO cells. *n* = 2 in each group. **b** Heatmap illustrating the eigenvector score and H3K9la and H3K18la modification signal intensities of B-to-A switched compartment regions in KO and nPKM2 cells (*n* = 4546). *n* = 2 in each group. **c** Integrative genomics viewer snapshot displaying the B-to-A compartment switching status, RNA-seq data and histone lactylation signals in KO and nPKM2 KGN cells. The red box indicates the gene body, and the vertical scale is shown on the left side of the box. *n* = 2 in each group. **d** Insulation score, CTCF, and H3K9la signal intensity of KO_Hi-C TAD boundary regions with or without PKM2 binding between KO and nPKM2 cells. The blue and red lines represent nPKM2 KO and parental KO cells, respectively. *n* = 2 in each group. **e** Heatmap of the differential peaks of the CTCF signal intensity distribution in KO and nPKM2 cells (1773 gained vs. 3986 lost sites). *n* = 2 in each group. **f** qPCR of *CTCF* expression levels in KO and nPKM2 KGN cells. *n* = 3 in each group. **g** Genome browser snapshot of RNA transcription at the *CTCF* locus in KGN and clinical granulosa cells. *n* = 3 in each group. **h** Genome browser snapshot of the PCOS-related *CPZ* locus showing Hi-C, insulation score of Hi-C, RNA-seq, interaction map, and CUT&TAG data in KO and nPKM2 cells. Hi-C interaction matrices are shown, with contact frequencies normalized to the data range. The raw read count values range from 0 to 40. The red bar shows the gene body, and the blue bar shows the *CPZ* locus interaction region in nPKM2 cells. *n* = 2 in each group. **i** Aggregate peak analysis plot for KO-, nPKM2- specific, and overlapping loops compared with each other. *n* = 2 in each group. **j** Proportion of nPKM2 cell-specific loops anchored with or without histone lactylation and overlapping with differential peaks associated with histone lactylation. **k** Genome browser snapshot of the *CYP17A1* locus showing RNA-seq, PKM2 binding, and H3K9la and H3K18la signals in KO and nPKM2 cells. The red box indicates the gene body, and the blue box indicates the *CYP17A1* loop anchor region. The vertical scale is shown on the left side of the box. *n* = 2 in each group

The nucleus houses chromatin, which is highly organized into a hierarchical 3D structure that is pivotal in the regulation of gene expression. Cis-regulatory elements, such as enhancers, are frequently distant from their target genes and approach gene promoters by bending and looping. Histone modifications and chromatin structural proteins can influence loop formation and topologically associated domain (TAD) boundaries to modify gene expression.^42,43^ We observed that histone lactylation is an active marker; however, how it influences global 3D chromatin organization and the regulation of gene expression remains unexplored. To investigate the effects of the nuclear accumulation of PKM2 and increased histone lactylation on chromatin structure, we used high-throughput chromatin conformation capture (Hi-C) to analyze 3D chromatin organization in nPKM2 and parental PKM2-KO cells (Supplementary Fig. 9a, b). First, we analyzed the status of the A/B compartment in nPKM2 cells and compared it with that in PKM2-KO cells. Globally, 4546 B compartments transitioned to A compartments across 7.05% of the genome, whereas only a small number of A compartments (1707) shifted to B compartments, covering 2.65% of the genome (Fig. 5a). Furthermore, the eigenvector score, which indicates the compartment status, was notably greater in nPKM2 cells in the upregulated gene regions (Supplementary Fig. 9c, d). Because B compartments represent a repressive status and A compartments represent active regions, these findings indicate that the chromatin status in nPKM2 cells is more active, which is consistent with our observation of a greater number of upregulated genes in nPKM2 cells. We also plotted H3K9la and H3K18la signals at the B-to-A compartment switch regions and observed stronger H3K9la signals in nPKM2 cells (Fig. 5b, c and Supplementary Fig. 9c), indicating that H3K9la contributes to the active chromatin state. Notably, the *ZNF467* locus underwent a significant B-to-A compartment switch with 78-fold increased expression (Fig. 5c), which is consistent with the findings in patients with PCOS.^44^

We further sought to understand the factors contributing to the more active chromatin status in nPKM2 cells at the TAD level. The TAD is the fundamental organizational unit of the genome, inside which DNA elements interact with each other more frequently and are separated by boundaries to preclude interactions with the neighborhood. Abundant TF and modulator binding at TAD boundaries is crucial for TAD arrangement. We categorized the Hi-C TAD boundaries on the basis of whether they overlapped with PKM2 binding sites and found that the overlapping boundaries had a smaller insulation score, suggesting weaker insulation among the TADs (Fig. 5d, upper panel). We then analyzed the CTCF occupancy and H3K9la signal strength at the TAD boundaries overlapping with PKM2 binding and observed reduced CTCF binding but enhanced H3K9la signals (Fig. 5d bottom panel). We examined the global CTCF binding status and confirmed a significant decrease in CTCF chromatin binding and gene expression in nPKM2 cells and patients with PCOS (Fig. 5e–g). Motif analysis further confirmed the enrichment of the CTCF motif in the lost binding regions but not in the gained binding regions (Supplementary Fig. 9e). The loss of CTCF binding can lead to TAD fusion events, which we observed in PCOS-related genes whose expression was upregulated due to TAD fusion. The PCOS-related gene *CPZ* is localized to a TAD boundary region in parental KGN cells. Nuclear accumulation of PKM2 results in TAD fusion events, leading to increased interactions between the *CPZ* locus and upstream genomic regions (Fig. 5h). To further elucidate the gene promoter and cis-regulatory interactions, we performed chromatin loop calling analysis and found that the nuclear localization of PKM2 was associated with a significant increase in chromatin loop formation (2567 loops compared with 1315 in Fig. 5i). These nPKM2-induced loop anchors were enriched in the histone lactylation signals (Fig. 5j, Supplementary Table 4 and Supplementary Fig. 9f). In conjunction with global 3D genome alterations, nPKM2 cells presented increased numbers of short-range loops and decreased numbers of long-range loops (Supplementary Fig. 9g), which is consistent with our previous findings of reduced TAD boundary insulation scores in nPKM2 cells. Notably, *CYP17A1*, a key gene in the androgen synthesis pathway, exhibited novel loop formation that brought its promoter into a distal regulatory element located 410 kb away, with concomitant increases in H3K9la and H3K18la signals (Fig. 5k), which may have contributed to the 49-fold increase in *CYP17A1* expression. In addition, we also found that the concentration of testosterone increased notably in nPKM2 cells. However, it had the opposite effect in the KO cells (Supplementary Fig. 9h), which suggests that androgen–PKM2–histone lactylation results in an epigenome-3D genome organization feedback axis.

Our findings suggest that the nuclear accumulation of PKM2, increased chromatin binding, strengthened H3K9la modification, and loss of CTCF binding collectively promote a more active chromatin state in nPKM2 cells, resulting in global changes in chromatin conformation and the consequent upregulation of genes related to PCOS.

### Arresting PKM2 nuclear retention with TEPP-46 rescues the PCOS-like phenotype

**Fig. 6:**
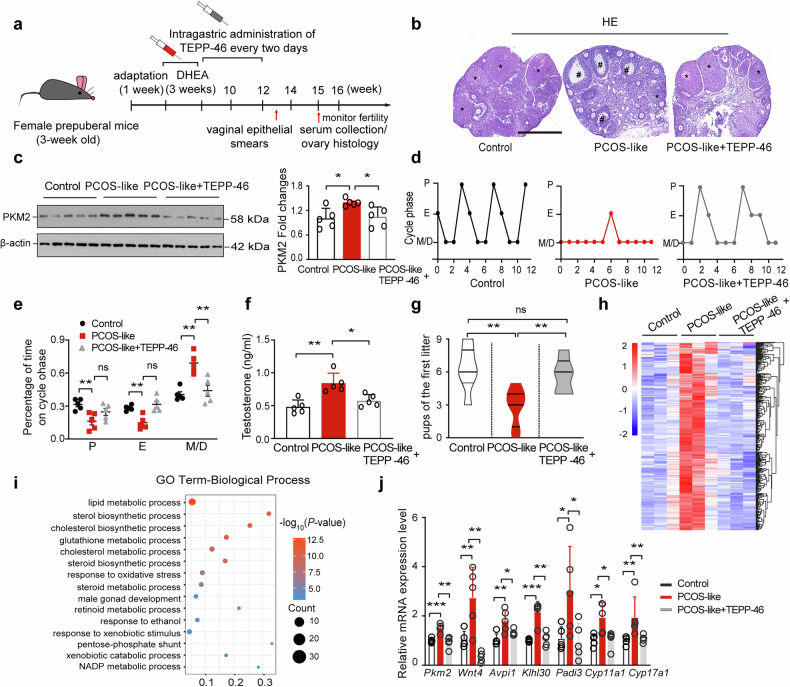
The PCOS-like mouse phenotype and upregulation of PCOS-related genes are reversed by TEPP-46. **a** Design of the TEPP-46 rescue assay using a PCOS-like mouse model. A PCOS-like model was constructed via dehydroepiandrosterone, and a TEPP-46 rescue experiment was performed according to the flow diagram. A series of assessments was performed on the control and treated mice. **b** Hematoxylin‒eosin-stained images of representative treated mouse ovaries. * indicates the corpus luteum; # indicates cystic follicles. Scale bar = 500 µm. *n* = 3 per group. **c** Western blotting showing PKM2 levels in ovarian granulosa cells (GCs) from control, PCOS-like, and PCOS-like+TEPP-46-treated mice (*n* = 5 per group). The data are presented as the means ± SEMs. The *p* values were determined via two-tailed unpaired Student’s *t* tests. * *p* < 0.05. **d**, **e** Continuous monitoring of the estrus stage in the control, PCOS-like, and PCOS-like+TEPP-46 groups (*n* = 5 per group). The data are presented as the means ± SEMs. The *p* values were determined by a two-tailed unpaired Student’s *t* test. ns indicates no significant difference, ** *p* < 0.01. **f** Testosterone levels were detected by ELISA in the three groups of mice (*n* = 5 per group). The data are presented as the means ± SEMs. The *p* values were determined by a two-tailed unpaired Student’s *t* test. * *p* < 0.05, ** *p* < 0.01. **g** Analysis of the number of pups born in the treated groups (*n* = 5 per group). The data are presented as the means ± SEMs. The *p* values were determined via two-tailed unpaired Student’s *t* tests. ns indicates no significant difference, ** *p* < 0.01. **h** Heatmap showing the expression levels of genes whose expression was elevated in GCs from the PCOS group and rescued in those from the PCOS + TEPP-46 group. (*n* = 3 per group). **i** Gene Ontology analysis of genes whose expression was upregulated in GCs from the PCOS group and rescued in those from the PCOS + TEPP-46 group. **j** Real-time qPCR analysis of the mRNA levels of *Pkm2*, *Wnt4*, *Avpi1*, *Klhl30*, *Padi3*, *Cyp11a1*, and *Cyp17a1* in GCs from control, PCOS, and PCOS + TEPP-46 mouse ovaries. (*n* = 5 per group). The data are presented as the means ± SEMs. The *p* values were determined by a two-tailed unpaired Student’s *t* test. * *p* < 0.05, ** *p* < 0.01, *** *p* < 0.001. The experiments were performed three times (**b**–**j**)

TEPP-46, a small molecule, induces Pkm2 tetramer formation, impairs Pkm2 accumulation, alters the glycolytic status of cancer cells, and xenografts of cancer cells are prevented from growing.^45^ Damasceno and colleagues demonstrated that nuclear Pkm2 exists as a dimer, whereas the tetrameric form of Pkm2 localizes to the cytoplasm.^46^ We constructed a PCOS-like mouse model as described previously.^8^ Mice were administered TEPP-46 every other day for 4 weeks immediately after dehydroepiandrosterone (DHEA) treatment (Fig. 6a). Notably, TEPP-46 treatment ameliorated the PCOS-like phenotype. The number of cyst-like follicles and corpora lutea found in TEPP-46-treated mice was similar to that in wild-type (WT) mice (Fig. 6b). The Pkm2 levels in the GCs of ovaries in the PCOS-like mouse group were significantly elevated, and TEPP-46 treatment restored the Pkm2 levels to those in WT mice (Fig. 6c). A hallmark of PCOS-like mice was disrupted estrous cycling, which was characterized by significantly increased time spent in metestrus and diestrus, and TEPP-46 rescued prolonged metestrus and diestrus in PCOS-like mice to control levels (Fig. 6d, e). Furthermore, the serum testosterone level and fertility of the TEPP-46-treated mice were also similar to those of the WT mice (Fig. 6f, g). From the 14th to 16th weeks, the weight curves indicated that the PCOS-like group had higher body weights than did the control group. Compared with no treatment, the administration of TEPP-46 to PCOS-like mice had no significant effect on body weight (Supplementary Fig. 10a).

To assess whether TEPP-46 could also help recover the PCOS-like transcriptome status back to normal, we analyzed ovarian GC RNA-seq data and revealed that the top 300 upregulated genes in PCOS-like mice were suppressed by TEPP-46 treatment, resulting in a transcriptional profile that resembled that of WT mice (Fig. 6h and Supplementary Table 5). The most variable genes were significantly enriched for Gene Ontology terms related to sterol biosynthesis, cholesterol biosynthesis, and response to oxidative stress (Fig. 6i), which are very similar to the transcriptome changes induced by nuclear PKM2. We confirmed the RNA-seq results via qPCR; in TEPP-46-treated mouse ovary GCs, PCOS-associated genes, such as *Pkm2*, *Wnt4*, *Klhl30*, *Avpi1*, *Padi3*, *Cyp11a1* and *Cyp17a1*, were downregulated to WT mouse levels (Fig. 6j). Additionally, TEPP-46 ameliorated dysfunctional metabolism, as assessed via glucose and insulin tolerance tests, O_2_ volume, and the respiratory exchange rate (RER) (Supplementary Fig. 10c-j). Our data suggest that the DHEA-mediated PCOS-like phenotype is likely achieved by affecting the expression and localization of *Pkm2* and that TEPP-46 can arrest Pkm2 nuclear retention and reduce its protein levels, thereby reversing PCOS-like transcriptomic states and the associated phenotype.

In conclusion, we elucidated the mechanism by which dysregulation of PKM2, a key metabolic enzyme, contributes to PCOS pathogenesis and highlighted the importance of histone lactylation on the 3D chromatin structure. The upregulation and nuclear translocation of *PKM2* in GCs leads to a shift in the histone lactylation landscape, which in turn promotes global changes in 3D genome organization in PCOS. PKM2-mediated histone lactylation results in a more active chromatin state, allowing certain distal enhancers to come into proximity to the promoters of key PCOS-related genes, such as *CPZ* and *CYP17A1*, through newly formed loops, thereby increasing the expression of these genes. The ability of the small molecule TEPP-46 to modulate PKM2 activity and rescue the PCOS-like phenotype suggests that *PKM2* represents a novel and promising therapeutic target for treating PCOS.

## Discussion

PCOS is a hormonal and metabolic disorder that is common among reproductive-age women and is characterized by hyperandrogenaemia, ovulatory dysfunction, and polycystic ovaries.^1,47^ The pathogenesis of PCOS is not well understood, despite its high prevalence and significant consequences for women’s health. Metabolic dysfunction is a core component of the PCOS phenotype. Approximately 60–80% of women with PCOS are obese, and obesity aggravates insulin resistance.^48^ A multinational cohort study revealed that women with PCOS had a significantly elevated risk of type 2 diabetes, with crude hazard ratios (HR, 95% CI) of 4.28 (3.98–4.60) in Denmark, 3.40 (3.11–3.74) in Finland, and 5.68 (5.20–6.21) in Sweden.^49^ The impairment of glucose homeostasis can interfere with normal follicular development. In a previous study, we demonstrated that the glycolysis gene *PGK1* reprogrammed gene expression in GCs and contributed to the onset of PCOS.^15^ Additionally, elevated expression of growth differentiation factor 8 (GDF8) caused ovulation failure by disrupting normal glucose metabolism in GCs.^50^ Given the frequent co-occurrence of glucose metabolism disorders in patients with PCOS, our objective was to elucidate how dysregulation of glucose metabolism, especially metabolic enzymes, leads to PCOS. Metabolites in FF significantly affect oocyte growth and ovulation.^51^ Increased serum lactate levels have been reported in patients with PCOS.^52,53^ We further revealed that higher levels of lactate were present in the plasma and follicular fluid of patients with HA-PCOS than in those with NA-PCOS. Furthermore, PKM2 contributed to genetic determinants of PCOS according to Mendelian randomization (MR) analysis of the causal associations between genetically predicted differences in glycolysis enzymes and the risk of PCOS. In addition, mass spectrometry was used to analyze GCs from patients with PCOS and controls to identify key enzymes, such as PKM2, that may drive PCOS pathogenesis. In the granulosa cells of PCOS patients, we observed significant upregulation and nuclear accumulation of this enzyme, a rate-limiting factor in the glycolytic pathway that generates lactate. Through epigenomic profiling, 3D-genome analysis, and animal modeling, we revealed that nuclear PKM2 induces PCOS-related gene expression through histone lactylation-mediated global changes in 3D chromatin conformation. Most importantly, blocking PKM2 nuclear translocation prevented the development of a PCOS-like phenotype in mice, highlighting a promising new therapeutic target for this disease.

In addition to its metabolic role in glycolysis, which occurs in the cytoplasm, PKM2 can translocate to the nucleus through allosteric regulation, thereby participating in the regulation of gene expression.^36,40^ The cytosolic tetrameric form of PKM2 is metabolically active, converting phosphoenolpyruvate to pyruvate, whereas the dimeric form translocates to the nucleus. Nuclear PKM2, a transcription coactivator, is responsible for gene regulation; it phosphorylates STAT3 to promote MEK5 transcription^54^ interacts with HIF1α to strengthen HIF1α binding to its target genes, and recruits p300 to reprogram gene expression.^55^ PKM2 can also regulate transcription by directly altering histone modifications.^36^ Upon EGFR activation, PKM2 translocates to the nucleus, forms a complex with β-catenin, and is recruited to the *CCND1* and *MYC* promoters. Moreover, it phosphorylates histone H3 at threonine 11 (T11), which displaces HDAC3 and facilitates H3K9 acetylation to activate gene expression.^36^ PKM2 phosphorylation of T11 of histone H3 is also associated with H3K4me3 upregulation.^56^ All these studies focused on investigating the regulatory role of PKM2 in gene expression at a single locus. However, the effect of nuclear PKM2 on global transcription remains largely unexplored. Our results indicated that nuclear, rather than cytoplasmic, PKM2 plays a pivotal role in inducing the PCOS-like phenotype and transcriptomic shift. In particular, the nuclear localization of PKM2 (nPKM2) induces the expression of several prominent PCOS-related genes, including *CYP11A1* and *CYP17A1*. TEPP-46, which promotes the formation of a tetramer of PKM2 and thereby blocks its nuclear translocation, effectively prevents the onset of the PCOS-like phenotype in mouse models. In this study, we also observed that H3T11 phosphorylation was elevated in nPKM2 cells via western blotting (data not shown). On the basis of the genome-wide upregulation of H3K9la, H3K18la, and H3K27la signals, we found that the binding sites of PKM2 in the genome were more closely associated with gained H3K9la peaks, contributing to more than 10% of the differential H3K9la signals. However, the precise mechanism through which PKM2 induces H3K9 lactylation requires further investigation. Because P300 possesses histone lactylation transferase activity^28^ and PKM2 can interact with P300,^55^ P300 may be involved in PKM2-mediated histone lactylation. On the other hand, several mitochondrial metabolic enzymes have been reported to translocate into the nucleus and are linked to epigenetic reconfiguration.^57,58^ For example, acetyl-CoA synthetase 2 (ACSS2) translocates into the nucleus upon glucose deprivation, and nuclear ACSS2 locally generates acetyl-CoA for H3 acetylation and gene expression.^59^ In our study, nuclear PKM2 likely catalyzes and increases nuclear lactate to serve as a substrate for histone lactylation. Furthermore, PKM2 binding signals and H3K9la peaks were enriched in the regulatory element regions of several PCOS-related genes and correlated with the upregulation of these genes. In brief, both our comprehensive whole-genome profiling and previous locus-specific studies highlight the crucial role of nPKM2 as an epigenetic regulator of gene expression. Notably, PKM2-mediated histone lactylation is a tremendously important contributor to PCOS; however, the possibility that lactate directly regulates gene expression and results in pathogenesis cannot be excluded. This specific regulatory mechanism needs further investigation.

Histone lactylation has been identified as a novel epigenetic modification,^25^ and it shares similarities with histone acetylation as an active histone marker associated with gene activation.^60^ For example, histone H4K12 lactylation has been linked to the activation of microglial proinflammatory genes in Alzheimer’s disease.^31^ Our study demonstrated that the upregulation of H3K9la and H3K18la signals is associated with increased gene expression, with a significant proportion of upregulated genes correlating with histone lactylation peaks. Given the pivotal role of histone acetylation in regulating enhancer‒promoter loop formation and 3D genome organization,^61^ we sought to explore how histone lactylation influences chromatin architecture. Our findings indicate that genome-wide upregulation of histone lactylation induced by nuclear PKM2 results in a more active chromatin conformation globally. Like H3K27 acetylation, which indicates active enhancer elements, we demonstrated that histone lactylation facilitated interactions between the cis-regulatory elements and the gene promoter, as well as between the cis-regulatory elements themselves. These findings suggest that histone lactylation can be utilized to predict cis-regulatory elements, such as enhancers. Moreover, in the entire promoter region of the upregulated genes, we observed significant enrichment of H3K18la and H3K9la signals, along with downregulation of H3K27ac (Supplementary Fig. 8d). These findings indicate that histone lactylation may involve a unique mechanism for regulating gene expression that is distinct from H3K27 acetylation. Additionally, these findings suggest that the histone lactylation signal can serve as a complementary indicator of active cis-regulatory elements. The binding of PKM2 and the enrichment of histone lactylation at TAD boundaries compromise CTCF binding, triggering TAD fusion events and facilitating the accessibility of cis-regulatory elements from neighboring TADs, thereby activating the expression of PCOS-related genes. CTCF, a pivotal architectural protein, plays a crucial role in maintaining the 3D conformation of chromatin by binding to TAD boundaries and forming chromatin loops, thereby preserving long-range interactions. Notably, CTCF, a key transcription factor, is critically involved in PCOS initiation and progression.^62^ We found that CTCF mRNA expression was reduced in nPKM2 cells and was downregulated in some patients with PCOS; most importantly, the *Ctcf* mRNA level was low in the GCs of PCOS-like mice, and TEPP-46 could partially rescue its expression levels (Supplementary Fig. 10b). Additionally, the CUT&Tag experiments demonstrated a global loss of CTCF chromatin-binding sites in nPKM2 cells, which was correlated with a decrease in the genomic insulation score and global upregulation of gene expression. Thus, investigating CTCF-mediated 3D genome organization in PCOS GCs may provide crucial mechanistic insights into this disease.

Although MR assumes no unmeasured confounding factors, our sensitivity analyses (MR‒Egger, weighted median) support the direct association of PKM2 with PCOS. Residual pleiotropy cannot be fully excluded, warranting future functional validation. While the consistency of PKM2 dysregulation across independent models (GCs cultures and DHEA-treated mice) supports its biological relevance, our human cohort was small. Future studies with larger, stratified cohorts (e.g., lean vs. obese PCOS patients) are needed to assess generalizability. While PKM2-KO models would be ideal, our current pharmacological and genetic inhibition data collectively support a pathogenic role of PKM2. Future studies utilizing ovary-specific PKM2-KO mice will be necessary to definitively establish its role in ovarian or oocyte function. While our findings demonstrate that TEPP-46 has an effective effect on the DHEA-induced model, we agree that its impact on genetic PCOS models (e.g., *Pten* knockout or *Cyp17a1* ectopic expression mice) remains untested and is an important research field.^63,64^ TEPP-46 rescued DHEA-induced PCOS phenotypes, but its applicability to other subtypes (e.g., genetic or insulin-dominant PCOS) requires further study. The long-term effects on the ovarian reserve and metabolic homeostasis also remain to be elucidated. In addition, TEPP-46 is a selective allosteric activator of PKM2 that has demonstrated promising preclinical results in modulating cancer metabolism and suppressing tumor growth. While TEPP-46 itself has not yet been tested in human clinical trials, accumulating evidence suggests that PKM2 is overexpressed in multiple human cancers, highlighting its potential as a therapeutic target. Furthermore, structurally related PKM2 modulators (e.g., ML-265) have shown early-stage translational potential,^65^ supporting the broader applicability of this drug. While promising in animal models, TEPP-46 requires further pharmacokinetic optimization before clinical evaluation.

**Fig. 7:**
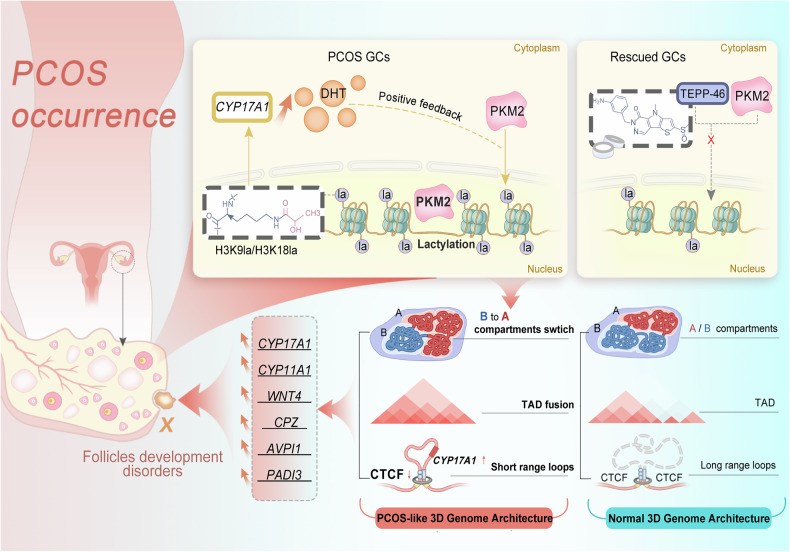
Proposed model for the effect of the PKM2/histone-lactylation module in promoting the occurrence of PCOS. The glycolytic enzyme PKM2 acts as a key nuclear factor that induces H3K9la and H3K18la modifications, thereby driving the remodeling of the three-dimensional genomic region architecture to regulate the gene expression of *CYP17A1*, *CYP11A1* and *WNT4*. This results in PCOS hyperandrogenism, which creates a positive feedback loop that leads to PCOS occurrence, and TEPP-46, a small molecule that inhibits PKM2 nuclear translocation, might act as a potential therapeutic target for the treatment of PCOS. The proposed model described above is drawn by Adobe Photoshop CC (version20, 2019)

In summary, our data support the notion that the androgen-induced nuclear accumulation of PKM2 promotes histone H3K9 and H3K18 lactylation. This PKM2-associated histone lactylation leads to changes in 3D genome organization, creating a more active chromatin state and enabling distal active elements to interact with the promoters of androgen synthesis- and PCOS-related genes. The androgen–PKM2–histone lactylation positive feedback loop in GCs may drive the pathogenesis of PCOS. Furthermore, the small molecule TEPP-46, which inhibits the nuclear translocation of PKM2 (Fig. 7), holds promise as a novel therapeutic target for the treatment of clinical PCOS patients.

## Materials and methods

### Ethics Statement

Ethical approval was obtained from the Ethics Committee of the International Peace Maternity and Child Health Hospital, Shanghai Jiao Tong University (Approval Numbers: GKLW 2015-42, GKLW 2017-71). All procedures were conducted in strict accordance with ethical guidelines. Written informed consent was obtained from all participants prior to their enrollment in the study. Animal experiments were conducted in accordance with the Guide for the Care and Use of Laboratory Animals at Fudan University (Approval ID: 2022JS-FCKYY-085). The animal procedures, which were designed to minimize suffering, were approved by the Institutional Animal Care and Use Committee.

### Study participants and GCs isolation or tissue collection

We used the Rotterdam criteria to diagnose PCOS in this study, which included patients from the outpatient population of the Reproductive Medicine Center at the International Peace Maternity and Child Health Hospital. Women undergoing in vitro fertilization (IVF) for tubal infertility or PCOS provided luteinized GCs. The follicular fluid (FF) that was drawn out when the oocytes were retrieved was spun at 1000 × *g* for 20 min. After the cell pellet was resuspended in PBS supplemented with 0.1% hyaluronidase, it was incubated at 37 °C for 20 min. Next, the GCs were further purified via Ficoll‒Paque (GE Healthcare Bio-Science) density gradient centrifugation. Patient characteristics are detailed in Supplementary Tables 6-9.

Fallopian tubes and uterine tissues were obtained from patients who underwent laparoscopy for cervical carcinoma in situ, benign ovarian cysts, or uterine leiomyoma. Samples were collected from patients who no longer required fertility and provided fully informed consent. Three uterine tissue samples adjacent to carcinoma in situ and three fallopian tube samples from hysterectomy patients (in the luteal phase) were used. No interindividual variability in PKM1/2 expression was observed.

### GWAS data sources

The GWAS data for glycolytic enzymes were sourced from a meta-analysis by Lotta LA et al.^66^ Data for PCOS patients were obtained from two independent sources: the UK Biobank (Sudlow C et al.)^67^ and a study by Day F et al.^11^ The original studies were approved by their respective institutional review boards or local ethics committees, and all participants provided informed consent. Genome-wide significant single-nucleotide polymorphisms (SNPs) associated with metabolic enzymes were identified from a publicly available meta-GWAS^66,68^ on the basis of a significance threshold of *p* < 5×10⁻⁸. This study included up to 174 metabolites and 86,507 individuals of European ancestry. SNPs were excluded on the basis of low minor allele frequency (MAF) or low imputation quality. The criteria were MAF < 2% or an imputation quality score < 0.3 for the Fenland and EPIC-Norfolk studies and MAF ≤ 1% or an imputed variants info score ≤ 0.4 for the INTERVAL study. Additionally, variants that failed the Hardy‒Weinberg equilibrium test (*p* ≥ 1×10⁻⁶) were excluded from the meta-GWAS. Finally, we removed variants with extreme statistical properties (absolute effect size >5, standard error >10 or <0) and all insertions and deletions (indels). Prior to association testing, mixed linear models were used to adjust for age, sex, and study-specific covariates.

To assess the impact of metabolic enzyme-encoding genetic determinants on PCOS, we leveraged two GWAS resources. This dataset was derived from the UK Biobank (imputed v3), a large prospective cohort. The analysis included 194,153 women of European descent, consisting of 436 patients with PCOS and 193,717 controls, selected from an available pool of 361,194 individuals. Phenotypic data were acquired via self-reports. A second dataset was sourced from a European meta-analysis,^11^ comprising 4,138 PCOS cases and 20,109 controls. For the meta-GWAS, cases were defined via two diagnostic criteria: the National Institutes of Health (NIH) criteria, which require both hyperandrogenism and ovulatory dysfunction, and the Rotterdam criteria, which require polycystic ovarian morphology in addition to at least two of the abovementioned traits.

### MR analysis

Preprocessing for Mendelian randomization (MR) analysis: A metabolic enzyme GWAS utilized a weighted z-score approach. To derive the effect estimates and standard errors for subsequent MR analysis, the z-scores were then transformed via the described method.^69^ The PhenoScanner database was used to identify instrumental variables (IVs) related to several traits and diseases (obesity, body mass index, and type 2 diabetes mellitus) that might serve as confounding factors, which were excluded via the pcf function in R software.^70^

Two-sample Mendelian randomization: Following the identification of genome-wide significant SNPs (*p* < 5 × 10^−8^) as instrumental variables (IVs), we conducted a series of two-sample Mendelian randomization analyses with key metabolic enzymes such as exposures and polycystic ovary syndrome (PCOS), utilizing the “TwosampleMR” package (version 0.5.7) in R software.^10^ Odds ratios (ORs) with 95% CIs are used to display the results. To reduce the impact of weak instrument bias, we determined the F statistic for each genetic variant (SNP), with an F statistic greater than 10 indicating a strong instrumental variable (IV).^11^ Our primary method for obtaining reliable effect estimates in two-sample MR analyses was inverse variance weighted (IVW). When directional pleiotropy is absent, IVW is the same as a weighted regression of the gene‒outcome association.^10^ To support the causal inference proposed by the IVW model, other MR approaches, such as MR‒Egger, weighted median, and weighted modes, have also been employed.^12^

We utilized *Cochran’s Q test* to indicate the possibility of horizontal pleiotropy and MR Egger regression to detect potential directional pleiotropy to assess the sensitivity of the results. When looking for outlier variables, we also employed the MR-PRESSO test, which stands for MR pleiotropy residual sum and outlier. Additionally, the individual Wald ratios for each SNP are shown in funnel plots, and the influence of the remaining SNPs recalculated via the IVW approach was presented via the leave‒one-out method.^13^

### 4D mass spectrometry analysis of clinical GC proteins

#### Sample digestion

The GCs were sonicated three times on ice in 8 M urea lysis buffer containing protease inhibitors. The supernatant was collected after centrifugation at 4 °C and 13,000 rpm for 12 min, and the protein concentration was determined with a BCA kit. Before being alkylated with 11 mM iodoacetamide for 15 min at room temperature in the dark, the proteins were reduced with 5 mM dithiothreitol for 30 min at 56 °C. After the sample was diluted with 100 mM TEAB (Sigma‒Aldrich, USA), the urea content decreased to less than 2 M after alkylation. Before the first digestion, which took place overnight with a trypsin-to-protein mass ratio of 1:50, a second digestion took place for 4 h with a trypsin-to-enzyme mass ratio of 1:100.

#### 4D mass spectrometry

A custom-made reversed-phase column (25 cm length, 75/100 μm i.d.) was used to load the tryptic peptides, which were dissolved in solvent A (a mixture of 0.1% formic acid and 2% acetonitrile). The following gradient was used to perform chromatographic separation on a Bruker Daltonics NanoElute UHPLC system at a steady flow rate of 450 nL/min: 70 min at 6% to 24% solvent B (0.1% formic acid in acetonitrile), 14 min at 24% to 35%, 3 min at 80%, and 3 min at 80% hold. To analyze the eluted peptides, a timsTOF Pro mass spectrometer was used in PASEF mode after they were ionized using a capillary source at 1.60 kV. Furthermore, mass spectrometry data were collected between 100 and 1700 m/z. Precursors with charge states of 0–5 were subjected to 10 PASEF-MS/MS scans each cycle and 30 s of dynamic exclusion to initiate fragmentation.

#### Database search

MaxQuant (v.1.6.15.0) was used to process the MS/MS data. This was performed against the human SwissProt database, which contains 20,422 entries and was augmented with a reverse decoy sequence. The database search was set up such that trypsin/P was used as the enzyme (which allowed for as many as two missed cleavages), the precursor mass tolerance was set to 20 ppm for the initial search and 5 ppm for the main search, and the fragment mass tolerance was set to 0.02 Da. The false discovery rate (*FDR*) was set to <1% at the peptide and protein levels. The GO annotations of proteins and other related analyses were performed via the PTM BIO (Hangzhou, China) protocol.

#### Cell culture, CRISPR–Cas9 gene editing, and generation of stable cell lines

The HEK293T and KGN cell lines, which are similar to human ovarian granulosa, were stored at Fudan University’s Institute of Reproduction and Development. DMEM (Gibco, NY, USA) was used for the culture of HEK293T cells, whereas DMEM/F12 (Gibco) was used for the growth of KGN cells. The 10% FBS (Gibco) supplement and 1% penicillin‒streptomycin were added to both media. Following previous methods, KGN cells were transfected with lentivirus harboring lentiCRISPRv2, together with control *lacZ* sgRNA or *PKM2* sgRNA, to establish knockout cell lines.^71^ After incubation for 48 h, the cells were treated with 1.5 µg/mL puromycin for an additional day. Single cells were then transferred to 96-well plates, and the clones were expanded and screened for relevant protein expression by immunoblotting. Successful editing at the target loci was confirmed via Sanger sequencing for each clone. Stable transgenic lines expressing either PKM2 or nuclear PKM2 were then generated by transducing cells with concentrated lentivirus in accordance with the manufacturer’s protocol (Genomeditech, Shanghai, China).

#### Plasmid construction

The full-length human PKM2 gene was cloned and inserted into the pFLAG-CMV-2 and pcDNA-HA vectors after amplification via appropriate primers via polymerase chain reaction (PCR). In addition, separate PKM2 CDSs were inserted into the pEGFP-C1 vector. Following the instructions provided by the manufacturer, HEK293T cells were transfected with plasmids via Lipo2000 (Invitrogen, Carlsbad, USA). qRT‒PCR and western blotting were used to confirm the mRNA and protein levels, respectively. The lactate sensor vector pcDNA-FiLa-Nuc was purchased from FR Biotechnology (Shanghai, China). We ligated the FiLa-Nuc DNA sequence and nuclear *PKM2* merged reporter gene tomato (nPKM2-tomato) into the pBoBi vector and expressed it in HEK293T cells to obtain virus particles. Then, the relevant cells were infected with the lentivirus. Images were captured under fluorescence illumination with a 20x objective lens via a confocal microscope (Digital Eclipse A1R + , Nikon).

#### Immunoprecipitation assays and western blotting

The NP-40 lysis buffer that was used to lyse the cells was supplied by Beyotime (Nanjing, China). The primary antibodies were incubated with the lysates overnight at 4 °C. To prepare for western blot analysis, antibody‒protein complexes were collected via protein A/G beads (Thermo Fisher Scientific). The samples were then washed four or five times with lysis solution. A protease inhibitor cocktail (YEASEN) was added to the lysis buffer before the cells were lysed for total protein detection. A BCA assay (Thermo Fisher Scientific) was used to measure the protein concentration. After being separated via SDS‒PAGE, proteins (20 μg per lane) were transferred to PVDF membranes. The membranes were first treated with primary antibodies at 4 °C for one hour after being blocked with 5% milk at room temperature. The membranes were treated with secondary antibodies conjugated with HRP after being washed three to five times with TBST. The ImageJ program was used to quantify the signals. The antibodies, chemicals, reagents, cell lines and organisms used in this study are described in Supplementary Table 10.

#### CUT&TAG and Hi-C

One hundred thousand cells were harvested for CUT&Tag analysis via the Hyperactive In Situ ChIP Library Prep Kit for Illumina (pG-Tn5; Vazyme Biotech) in accordance with the manufacturer’s guidelines. The cells were allowed to adhere to concanavalin A-coated beads and then resuspended in antibody buffer. The cells were subsequently treated with primary antibodies targeting H3K9la, H3K18la, H3K27la, PKM2, CTCF, H3K27ac, H3K4me3, and H3K27me3, together with their respective secondary antibodies. The samples underwent transposon activation and tagmentation, followed by DNA isolation, amplification, and purification to create a library. Library quantification was conducted via a Qubit fluorometric assay (Thermo Fisher Scientific). Sequencing was performed on an Illumina NovaSeq platform utilizing 150 base pair paired-end reads. KO and nPKM2 cells were grown for Hi-C assays in accordance with the manufacturer’s instructions. Crosslinking was conducted on 2–5 million cells utilizing 1% formaldehyde for 10 min at room temperature. Following permeabilization, the nuclei were subjected to digestion with 100 U of DpnII. The restriction ends were filled with biotinylated nucleotides and ligated. Following cross-link reversal, the DNA was purified and sheared to 400 bp. Biotin capture with streptavidin beads^72^ was used to enrich for ligation junctions prior to Illumina sequencing. Data analysis was performed as described below.

#### Quality control in sequencing

The raw fastq reads were trimmed via fastp (v0.23.1, https://github.com/OpenGene/fastp) tools with default parameters.^73^ In brief, the raw reads with Illumina next-generation sequencing adapters, low-quality bases and short lengths were filtered from the RNAseq, CUT&Tag, and Hi-C experimental libraries.

#### RNA-seq data processing

The clean fastq of the RNA-seq data was processed with the ENCODE-DCC RNA-seq pipeline (https://github.com/ENCODE-DCC/rna-seq-pipeline). Briefly, the clean fastq reads were aligned with the hg38 reference genome (https://www.encodeproject.org/files/GRCh38) via STAR software (STAR 2.5.1b),^74^ and the signal track of the uniquely mapped reads was generated via samtools (1.9.0),^75^ bedGraphToBigWig, and bedSort UCSC tools (https://hgdownload.soe.ucsc.edu). Gene expression values were quantified and calculated via RSEM software (v1.2.31).^76^ The raw count sum of each gene in all samples was less than 10, and those whose TPM values were less than 1 were removed. The edgeR R package (v4.2.2)^77^ was used to identify the differentially expressed genes (DEGs), followed by *FDR* (*P* value adjusted by BH methods) < 0.05 and an absolute log_2_fold change >= 1 as the threshold value. The GO enrichment terms of the DEGs were analyzed with the Metascape tool (https://Metascape.org/gp/index.html). The GSEA results of all the genes were analyzed via the clusterProfiler (v4.12.6) R package_,_^78^ and the significant terms were visualized via the GseaVis (https://github.com/junjunlab/GseaVis, v0.1.1) R package.

#### CUT&Tag data processing

The clean fastq of CUT&Tag data was analyzed with the “CUTTag_tutorial, (https://yezhengstat.github.io/CUTTag_tutorial/).” Briefly, the clean fastq was mapped to the hg38 reference genome via Bowtie2 (v2.5.1) tools with the “--end-to-end -- very-sensitive-- no-mixed-- no-discordant-- phred33 -I 10 -X 700” parameters.^79^ The mapped reads of PCR duplication were removed with Picard (v3.1.1, https://broadinstitute.github.io/picard/). The bam files of the unique mapped reads were subsequently converted to bed format with the “bedtools bamtobed” (v2.31.0) command.^80^ Next, SEACR (v1.3) software was used to call significant peaks against the experimental IgG control with “nonstringent” parameters, and the top 0.01 and top 0.1 peaks for PKM2 and all histone modification CUT&Tag binding sites were retained.^81^ DeepTools bigWigCompare (v3.5.4) was used to calculate the log_2_-fold change in histone markers between nPKM2-KO cells and parental KO cells, and the changes were visualized via a “plotheatmap” of deepTools. The quality and reproducibility between biological replicates were assessed via the mapped fragment size distribution and correlation analyses of the counts of mapped reads across the genome. Finally, the peaks were recentered to a fixed width of 500 bp, and differential peaks of histone markers were identified with a *p* value < 0.05 and |log_2_ Fold-change | >= 1 as the threshold via the DiffBind R package (v3.10.1).^82^

#### Hi-C data processing

We aligned the cleaned Hi-C FASTQ files to the hg38 reference genome via BWA-MEM (v0.7.17, -SP5M).^83^ Then, PCR duplicates were removed from the mapped reads with pairtools dedup (v1.1.0),^84^ and the “UU,” “UR,” and “RU” pair types were selected for further analysis. The 5, 10, 25, 40, 50, and 100 kb multiresolution matrices were generated via Juicer tools (v1.8.9) with a restriction site map of DpnII.^85^ Next, the multiresolution hic files were balanced and normalized into mcool files via Cooler software (v0.9.2).^86^ Finally, the stratum-adjusted correlation coefficient (SCC) of the Hi-C biological replicates was calculated via HiCRep (https://github.com/dejunlin/hicrep),^87^ and the biological replicates were combined for subsequent analysis.

The A and B compartment structures of chromatin were identified with cooltools (v0.5.4) tools by constructing a 50 kb Hi-C contact matrix with ICE normalization of each sample and calculating the frequent contacts with regions of the same type in the genome.^88^ The GC base content was used to orient eigenvectors as a phasing track and to assign genomic regions to the A or B compartment. The insulation score was calculated via cooltools with a 50 kb resolution ICE-normalized matrix with default parameters. Boundaries were selected from “boundary_strength_column,” and topologically associated domains were merged with adjacent regions via bedtools software. The chromatin loops were identified with Peakachu (v2.0.0)^89^ tools at 10 kb ICE-normalized resolutions. The predicted loops were selected using a probability score greater than 0.9. The differential loops were calculated with a probability score greater than 0.9 in sample A and less than 0.5 in sample B. The interaction of enhancer‒promoter contact was predicted with activity‒contact (ABC) model tools (v1.1.2, https://github.com/broadinstitute/ABC--enhancer‒gene‒prediction) in a 5 kb resolution Hi-C matrix with KR normalization in nPKM2 and KO cells with default parameters, as described previously.^90^

#### qRT‒PCR

TRIzol reagent (Invitrogen) was used to extract total RNA from several sources, including human granulosa cells, mouse ovarian tissue, and cultured cells, according to the manufacturer’s procedures. One microgram of RNA was transformed into complementary DNA via the RT Reagent Kit in conjunction with gDNA Eraser (RR047A; Takara, Japan). Equipment from Life Technologies (Carlsbad, USA), known as the QuantStudio 7 Flex, was used to perform the qRT‒PCR. Three independent analyses were performed on each sample. An internal control, actin, was used to normalize the expression levels of the target transcripts. The primers, sgRNAs and other sequences used in the study are listed in Supplementary Table 11.

#### Immunofluorescence staining

After being washed with phosphate-buffered saline (PBS), the cultured cells were preserved for 15 min in 4% paraformaldehyde (PFA). After 15 min of permeabilization with 0.5% Triton-100, the cells were blocked for one hour with 3% bovine serum albumin. After that, the samples were incubated with the assigned primary antibody overnight at 4 °C. Following several washes with PBS, the nuclei were counterstained with 4’,6-diamidino-2-phenylindole (DAPI) at room temperature and incubated with secondary antibodies labeled with Alexa Fluor 488 or 594 (Invitrogen, Carlsbad, USA) for one hour. LAS X image processing software and an SP8 confocal microscope were used to visualize the fluorescent signals.

#### PCOS-like mouse model and AAV vector injection

Female C57BL/6 J mice were acquired from Shanghai Model Organisms (Shanghai, China). The mice were three weeks of age and were allowed to acclimatize for one week before experimentation. To induce a PCOS-like phenotype, the mice received daily subcutaneous injections of DHEA at a dose of 0.06 g/kg body weight for a total of 21 days.^8,91,92^ At seven weeks of age, the mice in the TEPP-46 treatment group received TEPP-46 (1 mg/mL) by gavage for 4 weeks. Body weight was monitored once per week, and vaginal smears were collected daily for 16 consecutive days to monitor the estrous cycle. For histological evaluation, ovaries were fixed in 4% PFA, processed through a graded ethanol series, and embedded in paraffin. Sections of approximately 8 μm thickness were cut and subsequently stained with hematoxylin and eosin (H&E). Histomorphological observations were made with a Leica DM2500 microscope (Leica, Germany). The numbers and characteristics of cystic follicles and corpora lutea were evaluated, with confirmation provided by a professional pathologist. Construction of the mouse PKM2 AAV vector was carried out by OBiO (Shanghai, China). At six weeks of age, female C57BL/6 J mice were injected with the AAV vector directly into the ovaries under anesthesia. The mice were maintained under normal feeding conditions and subjected to relevant experiments before being sacrificed to harvest ovaries and other tissues. Ovarian protein expression was assessed by immunohistochemistry (IHC). The procedure involved antigen retrieval, incubation with primary and secondary antibodies, and the application of a detection reagent to visualize the bound primary antibody, following established protocols.^93^

#### Isolation of GCs from the ovaries

A 1:4 ratio of collagenase 4 (GIBCO #17104019) was used, and a digestive solution was prepared by dissecting the ovaries of the control, PCOS-like, and PCOS-like + TEPP-46 mice to remove extra fat. For 20 min, a 400 µL portion of the mixture was left to incubate at 37°C. Once the digestion was halted, 2 mL of DMEM/F12 (supplemented with 10% FBS and 1% penicillin‒streptomycin) was added. A total of three cycles of this procedure were performed for up to sixty minutes for digestion. Centrifugation at 1500 × *g* for 5 min at room temperature was performed after the cell suspension was filtered through a 70 µm mesh filter. Once the cells had been pelleted and resuspended for plate culture for 24 h, they were utilized for bulk RNA-seq or qPCR validation.

#### Transgene mouse lines and serum hormone detection

The R26-e (CAG-LSL-*Pkm2*-2A-tdTomato) mouse line was generated by Shanghai Model Organisms, and the AMH-Cre line was purchased from the Jackson Laboratory (CA, USA). R26-loxP-tdTomato mice were donated by Prof. Bin Zhou, as previously described.^94,95^ The TG PKM2 ^fl/fl^, AMH Cre^+^ PKM2 ^fl/fl^, and AMH Cre^+^ Tomato ^fl/fl^ lines were established by backcrossing the mice onto a congenic C57BL/6 J background. The animals were then subjected to a 12 h light/dark cycle. To obtain genomic DNA, mouse tails were subjected to lysis solution containing 0.1 mg/mL proteinase K at 65 °C for 3 h. The DNA was then denatured at 95 °C for 5 min and centrifuged at maximum speed for 5 min. Genomic PCR and DNA electrophoresis were used to genotype the mice. The mice were numbed, and blood was drawn from their hearts for the serum hormone test. Serum testosterone and AMH levels were determined via ELISA kits from LDN (Nordhorn, Germany) according to the manufacturer’s guidelines.

#### Whole-mount microscopy

After being anesthetized, the mice were subjected to transcardial perfusion with 10 mL of cold PBS to exsanguinate the ovarian tissue. The ovaries were immersed in 4% PFA at 4 °C for 20 min to 1 h, contingent upon tissue size, and subsequently rinsed three times with PBS. Whole-mount fluorescence images were acquired via a Zeiss stereoscope (AxioZoom V16), as previously described.^96^

#### Glucose tolerance test (GTT) and insulin tolerance test (ITT)

Twelve hours before the GTT and four hours before the ITT, the mice were fasted. An Accu-Chek Performa blood glucose meter (Roche, Los Gatos, USA) was used to monitor blood glucose levels via blood samples taken from the tail vein. After the animals’ glucose levels were measured at baseline, they were given either insulin (1000 − 1 IU/g BW) for the insulin tolerance test or glucose (G6152, Sigma‒Aldrich) (2 mg/g body weight) intraperitoneally for the glucose tolerance test. At 15, 30, 45, 60, and 120 min, blood glucose levels were checked.

#### Assessment of metabolism in cells and mice

The appropriate treatments were applied to KGN cells after they were seeded in 6-well plates. The levels of glucose (GAGO20, Sigma‒Aldrich) and lactate (K607, BioVision, Milpitas, CA, USA) were measured in the culture medium supernatants following the manufacturer’s recommendations. To measure the ECAR and OCR of KGN cells, we followed a slightly modified version of the procedure reported by Chakraborty et al.^97^ A Seahorse XFe96 Analyzer from Agilent Technologies (Santa Clara, CA, USA) was used. CLAMS (Columbus Instruments, Columbus, USA) was used to evaluate the physical and metabolic phenotypes of the control, PCOS-like, and TEPP-46 intervention groups in vivo. Housed in conditions that allowed for unfettered access to food and water, the mice were subjected to a 12-h light/dark cycle and maintained at a constant temperature of 22 °C. After 24 h of adaptation, we measured each mouse’s food intake, CO_2_ and O_2_ volumes, RER, and energy expenditure.

#### Bulk RNA-seq

We used an Agilent 2100 TapeStation to evaluate the quality of the RNA recovered from mouse ovarian GCs after TRIzol isolation; RNA integrity numbers greater than 8.0 were chosen for additional study. We used SuperScript II reverse transcriptase (Thermo Fisher Scientific) according to the manufacturer’s instructions to synthesize double-stranded cDNA from samples treated with TRIzol LS. The cDNA was subjected to 15 rounds of PCR amplification, resulting in a final elution volume of 24 μL. Following the instructions provided, a 100 ng cDNA sample was prepared via an Illumina DNA Prep Kit (20018704, Illumina). To sequence the samples via 50-base-pair paired-end (PR50) reads, they were pooled and loaded onto an Illumina NovaSeq 6000 after DNA concentration normalization. The exclusion of low-quality samples was achieved by postmapping quality checks. To ensure that the sequencing reads were of high quality, we used FastQC v0.11.9 and MultiQC v1.12. The STAR aligner (v2.7.1) with default parameters was used to align the RNA-seq reads to the GENCODE GRCm39 mouse transcriptome after trimming with the Illumina DRAGEN FASTQ toolbox (v1.0.0). To analyze differential gene expression, the STAR aligner was used with the “--quantMode GeneCounts” option to create raw gene counts. Normalization and batch-effect correction were performed after quality evaluation of the read counts. The differential expression analysis was conducted via DESeq2 (v1.34), and genes with low expression were filtered out via a cutoff of ≤100 raw counts across samples. We performed differential gene expression analysis and generated z-score heatmaps via normalized count data from DESeq2. Z-score heatmaps were created via normalized counts, principal component analysis (PCA) plots were generated via variance-stabilizing transformed (VST) data, and sample relatedness was assessed via Spearman correlation.

#### Metabolic flux

[^13^C₆]-Glucose (99%) was acquired from Cambridge Isotope Laboratories. The same number of cells in the control, KO, and nPKM2 groups were seeded in 10 cm dishes. After reaching suitable confluence, the cells were washed twice with PBS and then grown for six hours in DMEM (Gibco, #11966025), which did not contain any glucose but contained 10% FBS, 1% penicillin‒streptomycin, and 24 mM glucose (with a 1:2 ratio of [^13^C₆]-glucose to unlabeled glucose). After the cell pellets were washed with PBS, the metabolites were removed from the flash-frozen pellets. The pellets were lysed through homogenization in ice-cold methanol (1 mL) supplemented with 10 µg/mL myristic-d27 acid (internal standard). After vortexing for 20 s, the cell debris was pelleted by centrifugation at 15,000 × *g* for 10 min at 4 °C. Overnight lyophilization of a 700 µL aliquot of the supernatant from a Labconco CentriVap with a –80 °C cold trap was performed. In two steps, the dried metabolite extracts were derivatized. The samples were dissolved in 50 µL of methylpyridine solution (20 mg/mL), vortexed for 2 min, sonicated on ice for 6 min, and then incubated at 37 °C for 1.5 h. After adding 50 µL of MTBSTFA, the mixture was incubated at 37 °C for another hour. After derivatization, the samples were centrifuged at 15,000 × *g* for 12 min, and 50 µL of the supernatant was transferred to a GC vial for analysis. GC‒MS analysis was performed on an Agilent 5977B GC‒MSD instrument equipped with an HP‒5MS column (30 m × 0.25 mm, 0.25 µm film thickness; 19091S‒433). These temperatures were 260 °C and 280 °C for the injector and MSD transfer lines, respectively. The oven temperature program was as follows: the temperature was maintained at 70 °C for 2 min and then increased to 180 °C at a rate of 7 °C per minute, 250 °C at a rate of 5 °C per minute, and 310 °C at a rate of 25 °C per minute. The temperature was then maintained at 310 °C for 15 min. The quadrupole and source temperatures were 150 °C and 230 °C, respectively. Data acquisition and analysis were performed with Agilent MassHunter software (Acquisition v.B.07.04.2260; Qualitative Analysis v.B.07.01SP1). The air-dried protein pellets were dissolved in 0.2 M KOH at room temperature, and the protein concentrations were quantified via the Bradford assay.

### Statistical analysis

Statistical analyses were performed with Excel and GraphPad Prism 9.0. All the experimental results were reproducible, and the number of independent replicates for each experiment is provided in the corresponding figure legend. The data represent one of three repeats. For the animal experiments, the sample size (n) was determined on the basis of established standards from prior literature in this field.^8,10^ The data are shown as the means ± SEMs. We used two-tailed unpaired *t* tests, one-way ANOVA, or two-way ANOVA with Tukey’s post hoc test for statistical analysis. The significance is denoted as follows: *p < 0.05, ** *p* < 0.01, *** *p* < 0.001 and ns indicates no significant differences.

## Supplementary information

Supplementary_Materials

Supplementary Table 1-11

Uncropped films of Western blots

resource data

## Data Availability

All raw sequence data (Cell line RNA-seq, Clinical GCs RNA-seq, CUT&Tag, and Hi-C) reported in this paper have been deposited in the Genome Sequence Archive (GSA) at the Beijing Institute of Genomics (BIG) Data Center, Chinese Academy of Sciences, under the BioProject ID PRJCA027826 (https://ngdc.cncb.ac.cn/bioproject/browse/PRJCA027826). The MS proteomics data have been deposited in the ProteomeXchange Consortium (http://proteomecentral.proteomexchange.org) through the iProX partner repository with the dataset identifier PXD068345. The raw metabolomic data reported in this paper have been deposited in the OMIX, China National Center for Bioinformation / Beijing Institute of Genomics, Chinese Academy of Sciences (https://ngdc.cncb.ac.cn/omix: accession no. OMIX012247). All data supporting the findings of this study are available in the main text and its supplementary information.
