# Supplementary material for: Pyruvate kinase M2-mediated histone lactylation alters three-dimensional genomic architecture in polycystic ovary syndrome
Source: Signal Transduct Target Ther. 2025 Nov 19;10:376. doi: 10.1038/s41392-025-02468-5 (PMC12627565; doi:10.1038/s41392-025-02468-5)
Supplement: Supplementary file 1 — Supplementary_Materials [file 41392_2025_2468_MOESM1_ESM.docx]

Supplementary Materials for

**Pyruvate Kinase M2 (PKM2)-mediated Histone Lactylation Alters Three-dimensional Genomic Architecture in Polycystic Ovary Syndrome**

Chuanjin Yu^1,2,6#^, Tingting Liu^4#^, Yishu Wang^1,2,3,6#^, Xinghui Guo^4#^, Yujie Chen^5^, Yifan Zhao^3^, Xia Liu^3^, Weiwei Huang^1,2,6^, Shuoyang Zhao^1^, Jiaying Mo^7^, Hongtao Hu^8^, Pingping Lv^9^, Xiaotao Wang^1,2,6^, Zuwei Yang^1,2^, Jiexue Pan^1,2,6^, Guolian Ding^1,2,6^, Jianzhong Sheng^9^, Xinmei Liu^1,2,6*^, Hongbo Yang^1,2,6*^, He-Feng Huang^1,2,3,6,9^^*^

Correspondence to: He-Feng Huang (huanghefg@hotmail.com), Hongbo Yang (hongboyang@fudan.edu.cn), Xinmei Liu (liuxinmei@fudan.edu.cn)

**This PDF file includes:**

Supplementary Figures 1 to 10

Supplementary Tables 1 to 11

Uncropped films of Western blots

Resource data


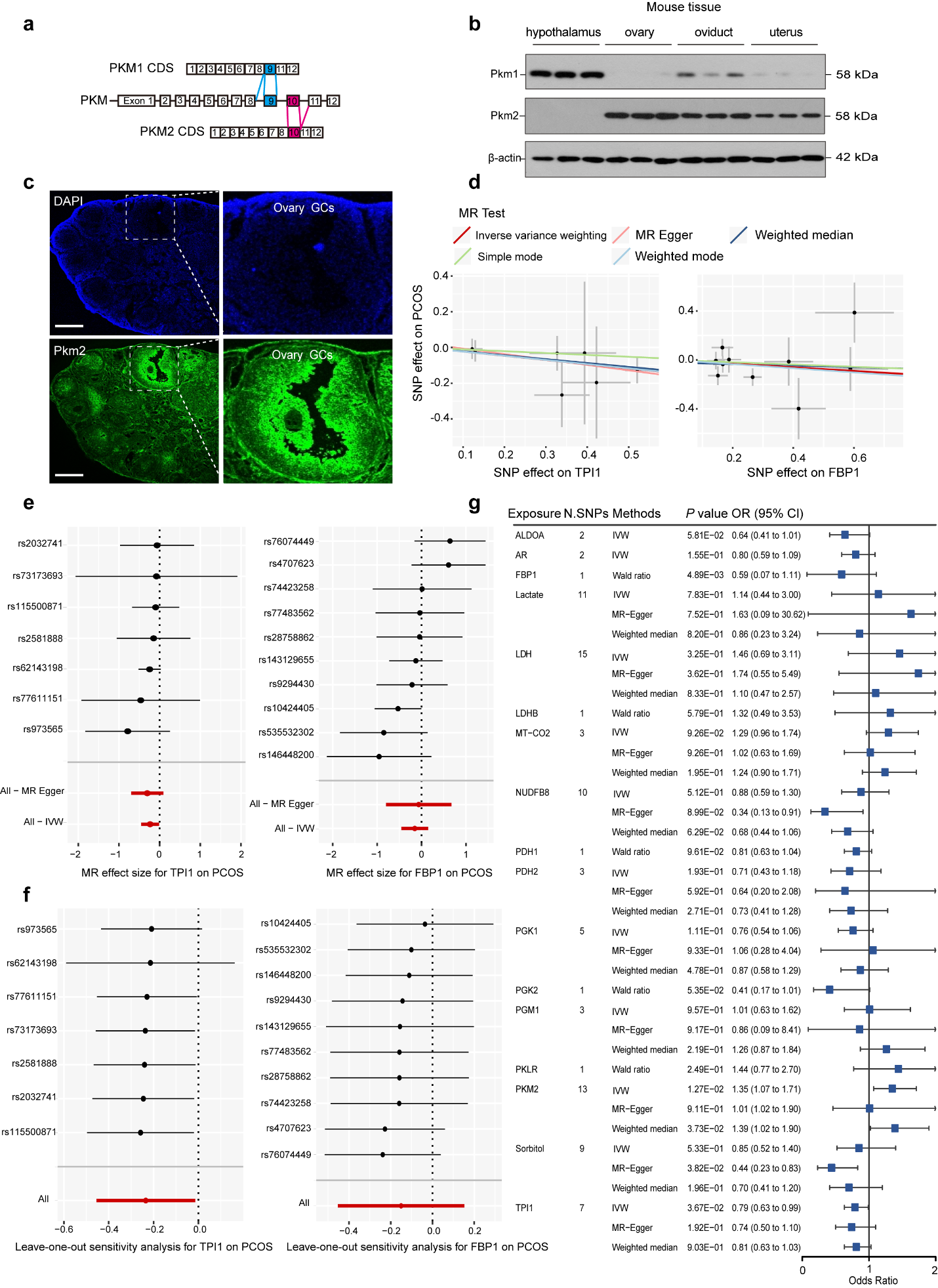


Supplementary Fig. 1

**The expression pattern of PKM1 and PKM2 in mouse reproduction related tissues and evaluated associations of key factors involved in glycolysis with PCOS using MR approach. a** In green and red, respectively, depict the mutually exclusive exons of *PKM1* (exon 9) and *PKM2* (exon 10) as indicating the *PKM* alternative splicing. **b** Western blot showing Pkm1 and Pkm2 levels in mouse reproductive-related tissues. (*n* = 3 per group). **c** Immunofluorescence data for Pkm2 expression in mouse ovaries. Scale bars = 100 µm. (*n* = 3 per group). **d** Scatter plots showing estimates of the risk of PCOS according to significant metabolic enzyme TPI1 and FBP1 exposures in glycolysis. **e** Forest plots of TPI1 and FBP1 illustrating the genetically predicted MR effect sizes of individual and combined SNPs for each exposure–outcome pair. **f** IVW MR regression results for the leave one SNP out analysis in the sensitivity analysis of TPI1 and FBP1. TPI1, triosephosphate isomerase 1; FBP, fructose 1,6-bisphosphatase; PCOS, polycystic ovary syndrome; IV, inverse variance; IVW, inverse variance weighted; MR, Mendelian randomization; SNP, single-nucleotide polymorphism. **g** Forest plot demonstrating the evaluated associations of key factors involved in glycolysis with PCOS using MR. The effect estimates represent the ORs for PCOS per 1-SD increment of the exposure. The error bars represent 95% Cis (confidence interval). IVW, inverse variance weighted; SNP, single-nucleotide polymorphism; LL, lactate levels; MR, Mendelian randomization; N. SNPs, the number of SNPs; OR, odds ratio. The associated genes in the study are in the following: ALDOA; AR; FBP; LDH; LDHB; MT-CO2; NUDFB8; PDH1; PDH2; PGK1; PGK2; PGM; PKLR; PGM1; TPI1.


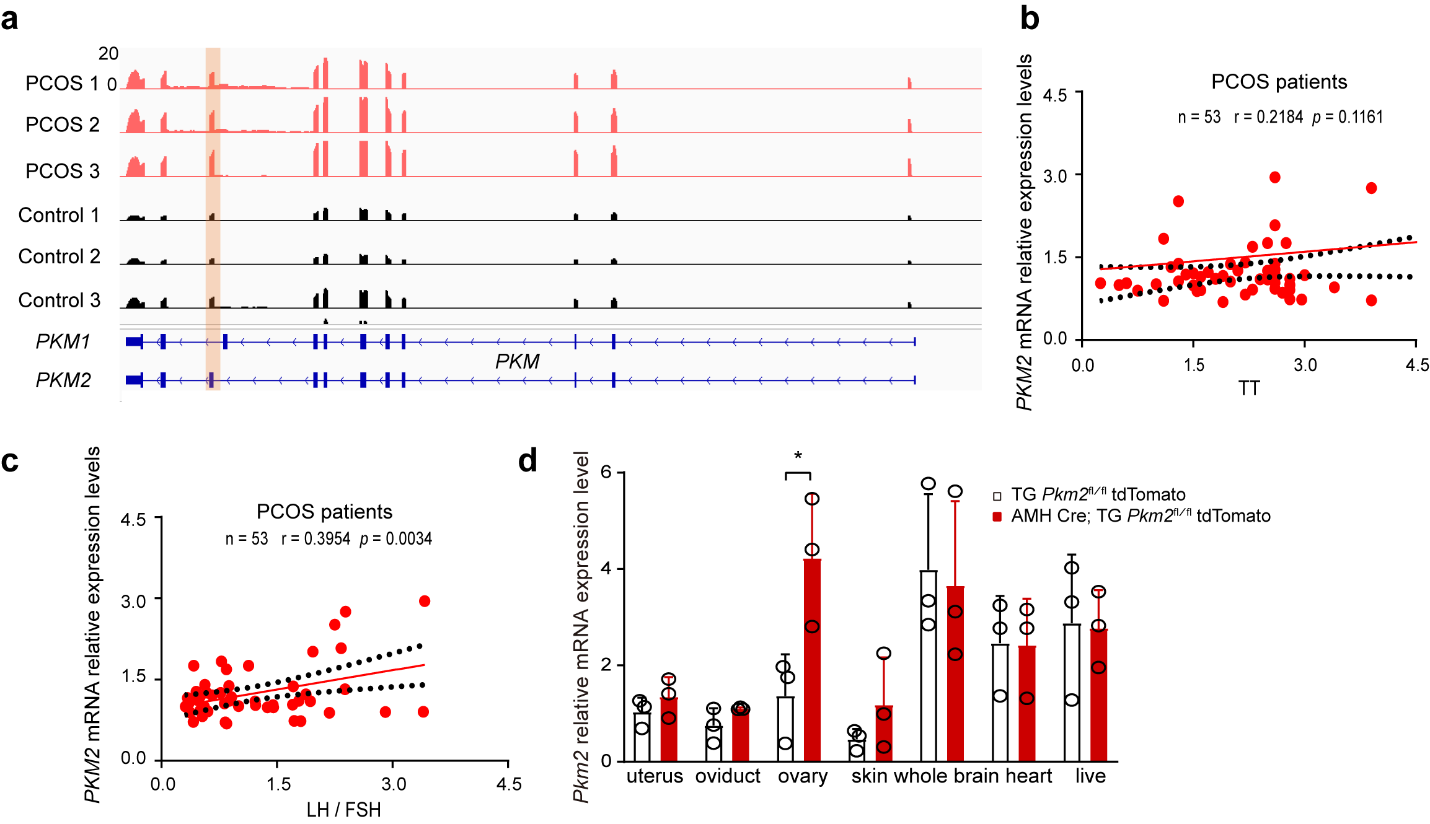


Supplementary Fig. 2

**Association of *PKM2* mRNA expression with clinical patient hormone levels and *Pkm2* expression level in main transgene mice tissues.** **a** Genome browser snapshot showing high expression levels of *PKM2*, but not of *PKM1*, in RNA-seq data for granulosa cells (GCs) from clinical PCOS than in Control (*n* = 3, per group). Yellow bar indicates exon 10 of the *PKM* locus. **b** Correlation of *PKM2* mRNA expression with serum testosterone in PCOS patients (*n* = 53). Data were analyzed by the Pearson (r) correlation and two-tailed Student’s *t*-test. **c** Correlation of *PKM2* mRNA expression with serum LH/FSH (*n* = 53). Data were analyzed by the Pearson (r) correlation and two-tailed Student’s *t*-test. *p* value < 0.05 indicates significance. **d** Different tissues of relative *Pkm2* levels were checked in TG- *Pkm2*^fl^ / ^fl^ tdTomato and TG-Tomato^fl^ / ^fl^;AMH Cre^+^ mice. (*n* = 3, per group). Data represented as the mean ± SEM. The *p*-values were determined by Two-tailed unpaired Student’s *t*-test. * *p* < 0.05. Experiments were performed three times.


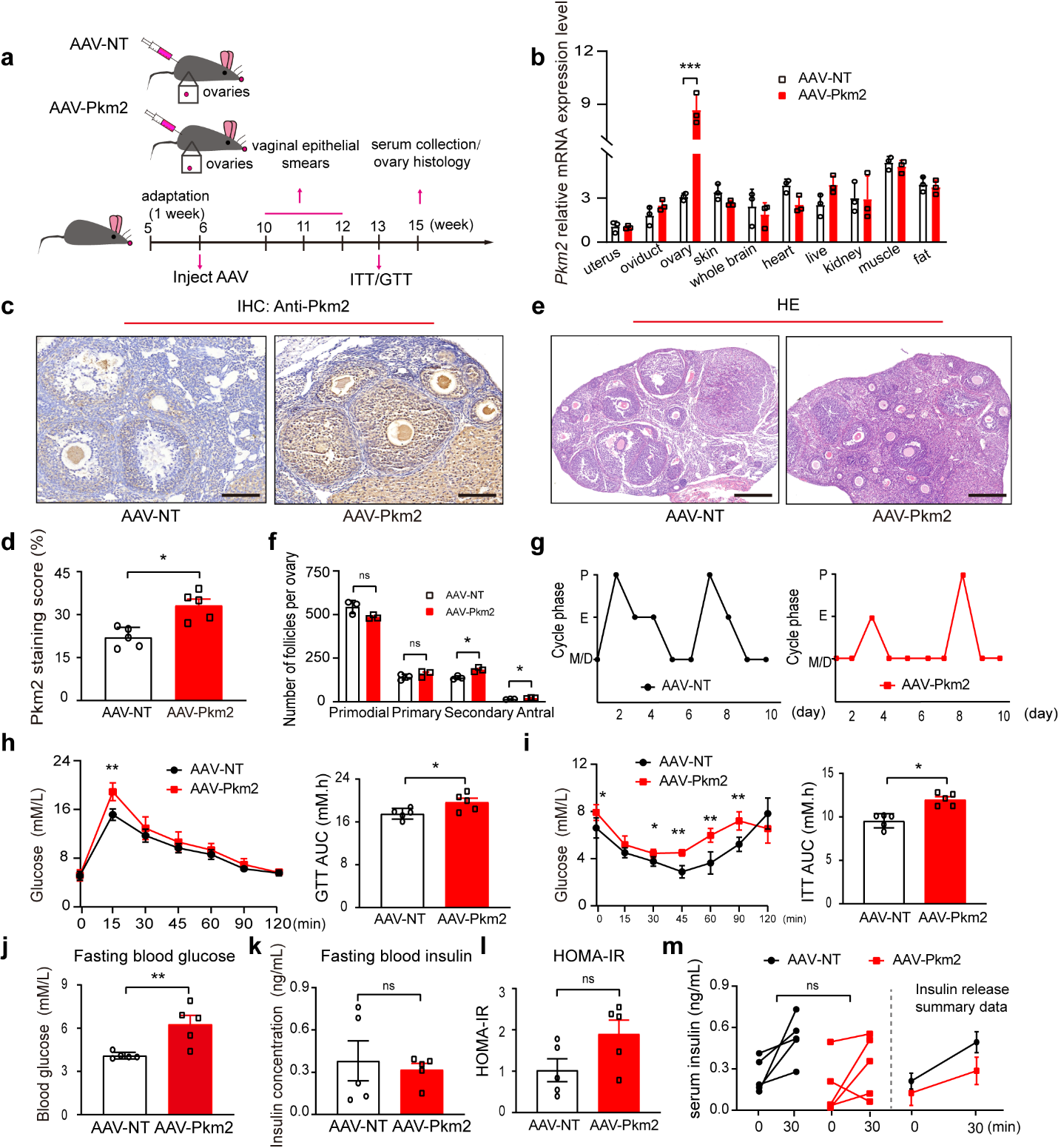


Supplementary Fig. 3

***Pkm2* ectopic expression in mice ovaries using adenovirus-associated virus (AAV) drives PCOS-like phenotype and metabolic dysfunction.** **a** The design of AAV-mediated *Pkm2* expression in mice ovaries. AAV injection and relevant treatments were performed according to the flow diagram. A series of assessments was made using the control and treated mice. **b** The fold changes of *Pkm2* expression levels were detected in different tissues of the AAV-Pkm2 treated mice (*n* = 3, per group). Data represented as the mean ± SEM. The *p*-values were determined by a Two-tailed unpaired Student’s *t*-test. *** *p* < 0.001. **c** Images of IHC staining with the Pkm2 in the ovaries of mice treated with AAV-NT- and AAV-PKM2. Scale bar = 100 µm. **d** Histogram of the PKM2 IHC staining scores showing the mean ± s.d. for the relevant groups (*n* = 5). Th *p*-values were determined by Two-tailed unpaired Student’ *t*-testst. * *p* < 0.05. **e** Representative images of hematoxylin and eosin-stained mouse ovary sections from the AAV-NT- and AAV-*Pkm2*-treated mice (*n* = 3, per group). * shows corpus luteum. Scale bar = 100 µm. **f** Number of follicles (including primordial, primary, secondary, and antral follicles) per ovary (*n* =3, per group). Data represented as the mean ± SEM. The *p* values were determined by Two-tailed unpaired Student’s *t*-test. ns showed no significant difference, * *p* < 0.05 **g** Continuous monitoring of the estrus stage in the AAV-NT- and AAV-*PKM2*-treated mice groups (*n* = 5). **h** Glucose tolerance test for the AAV-NT- and AAV-*PKM2*-treated mice (*n* = 5). **i** Insulin tolerance test for the AAV-NT- and AAV-*Pkm2*-treated mice (*n* = 5). Data represented as the mean ± SEM. The *p* values were determined by Two-tailed unpaired Student’s *t* test. * *p* < 0.05, ** *p* < 0.01. **j** Fasting blood glucose test for the AAV-NT- and AAV-*Pkm2*-treated mice (*n* = 5). Data represented as the mean ± SEM. The *p*-values were determined by a Two-tailed unpaired Student’s *t*-test. ** *p* < 0.01. **k** Fasting blood insulin test for the AAV-NT- and AAV-*PKM2*-treated mice (*n* = 5). Data represented as the mean ± SEM. The *p*-values were determined by a Two-tailed unpaired Student’s *t*-test. ns showed no significant difference. **l** Homeostasis model assessment-insulin resistance (HOMA-IR) test for the AAV-NT- and AAV-*PKM2*-treated mice (*n* = 5). Data represented as the mean ± SEM. The *p*-values were determined by Two-tailed unpaired Student’s *t*-test. ns showed no significant difference. **m** Serum insulin levels after 30 min of fasting and fold changes in serum insulin levels after glucose injection (*n* = 5). Data represented as the mean ± SEM. The *p*-values were determined by a Two-tailed unpaired Student’s *t*-test. ns showed no significant difference. Experiments were performed three times.


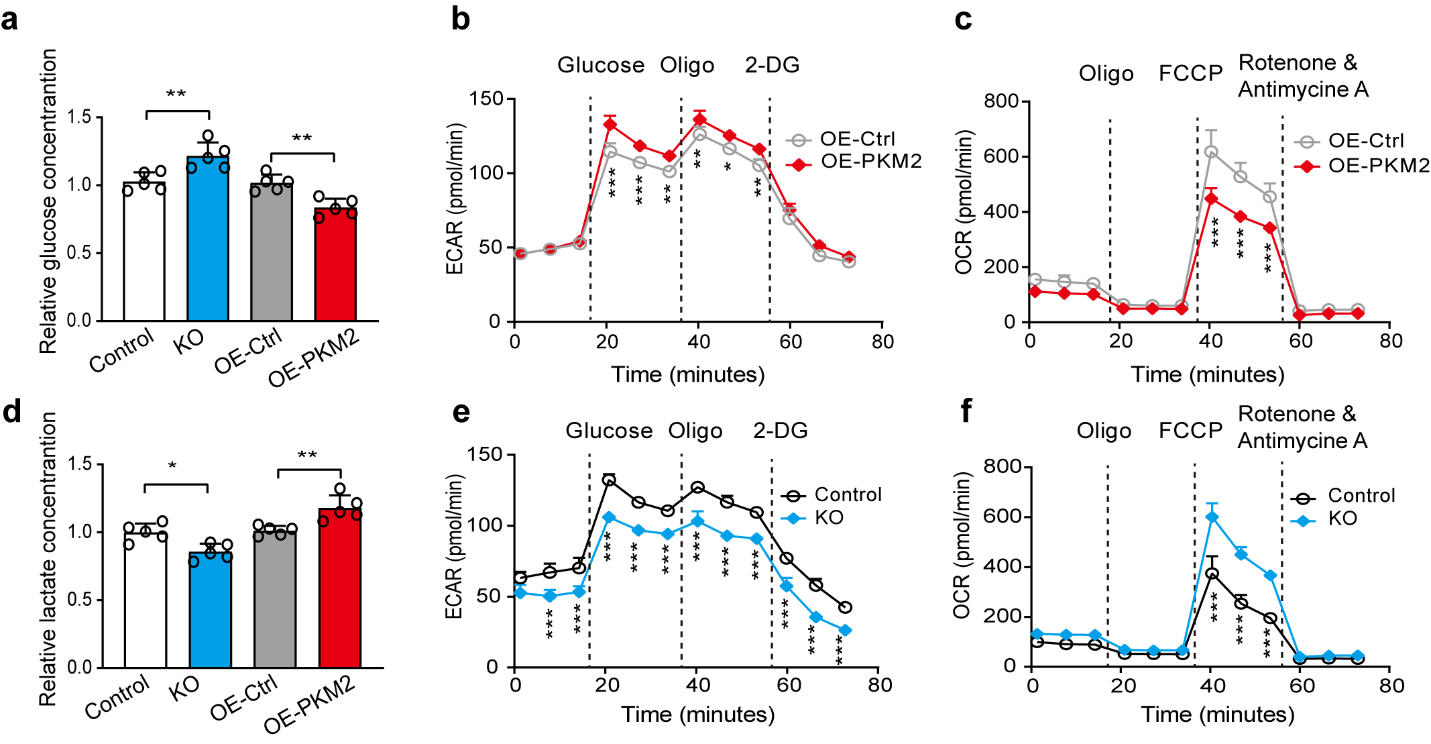


Supplementary Fig. 4

**PKM2 reprograms cells metabolism in KGN cells.** **a** Glucose levels in KO (PKM2 Cas9-knockout cells) and OE-PKM2 (PKM2-ectopic expressing) KGN cells compared with those in control KGN cells (*n* = 5). Data represented as the mean ± SEM. The *p* values were determined by Two-tailed unpaired Student’s *t*-test. ** *p* < 0.01. **b**-**c** ECAR and OCR in OE-PKM2 and control KGN cells (*n* = 5). Data represented as the mean ± SEM. The *p*-values were determined by Two-tailed unpaired Student’s *t*-test. * *p* < 0.05, ** *p* < 0.01, *** *p* < 0.001. **d** Comparing lactate levels in KO and OE-PKM2 cells with those in control (*n* = 5). Data represented as the mean ± SEM. The *p*-values were determined by Two-tailed unpaired Student’s *t*-test. * *p* < 0.05, ** *p* < 0.01. **e**-**f** The ECARs and OCRs assay were checked in KO and control cells (*n* = 5). Data represented as the mean ± SEM. The *p*-values were determined by a Two-tailed unpaired Student’s *t*-test. *** *p* < 0.001. Experiments were performed three times.


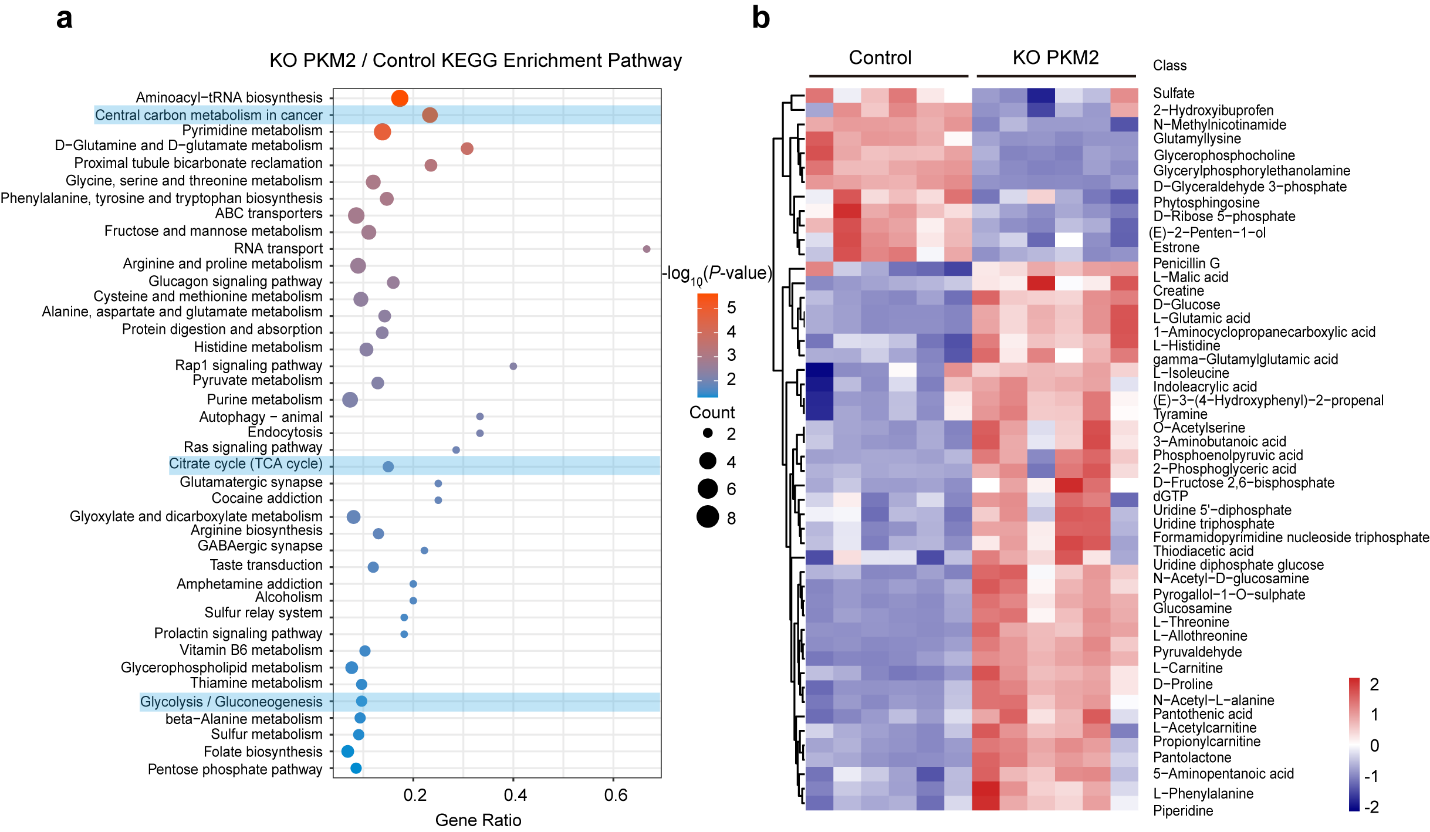


Supplementary Fig. 5

**Signaling pathway of metabolic flow and metabolic products regulated by *PKM2*.** **a** KEGG enrichment of signaling pathways in PKM2 knock out (KO) cells compared with control. **b** Global changes in the metabolic products in PKM2 KO cells compared with control, *n* = 6 per group.


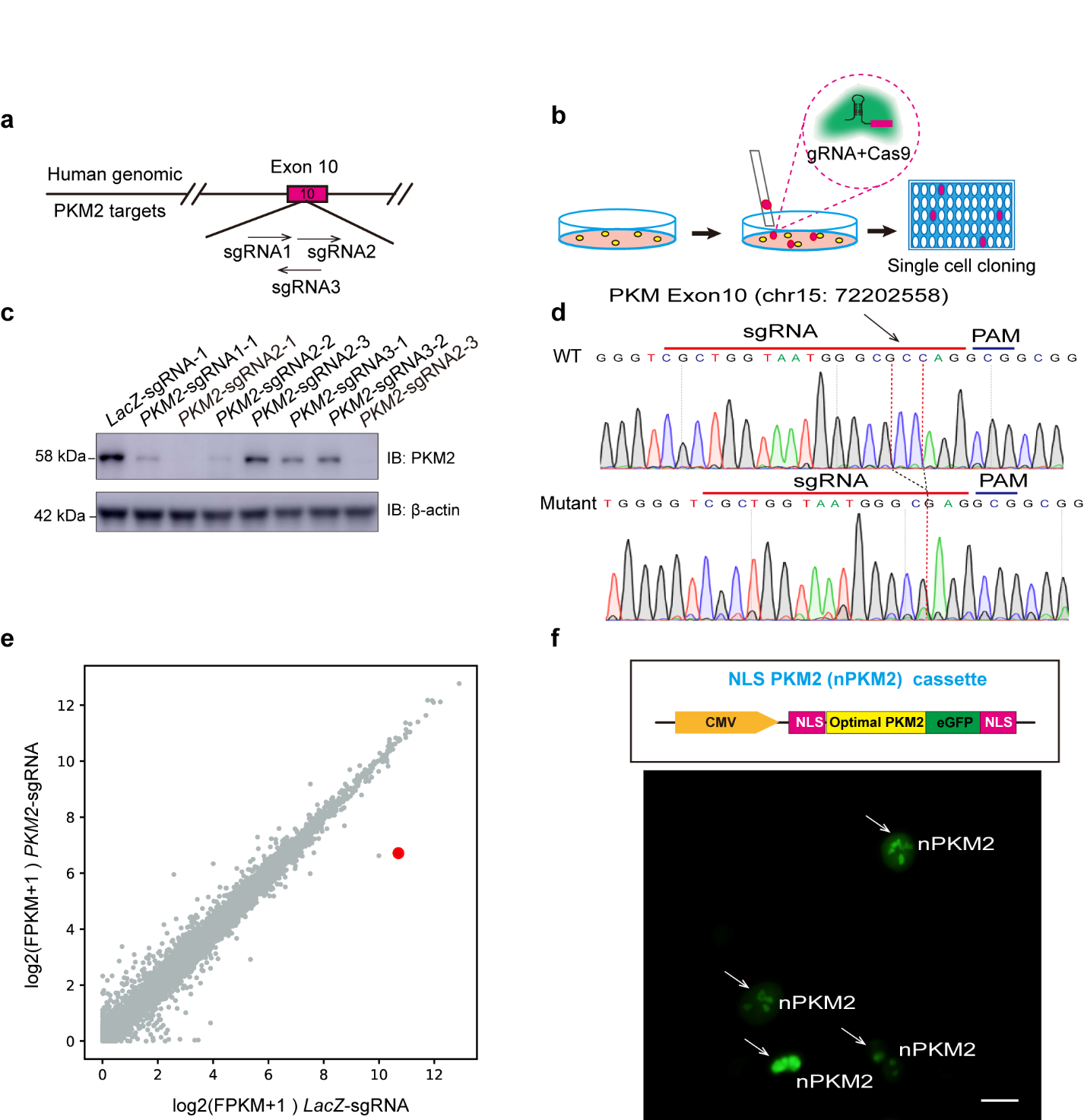


Supplementary Fig. 6

**Screening *PKM2* knock-out clones generated using the CRISPR/Cas9 system.** **a** Schematic showing the three gRNA target sites in the exon 10 of *PKM*. **b** Schematic showing Cas9-mediated knockout of target gene and single-cell cloning in 96-well plates. **c** Western blot showing PKM2 levels in independent PKM2 knock-out single-cell clones. **d** Sanger sequencing chromatograms indicating nucleotide information for the *PKM2* locus in CRISPR/Cas9-engineered single-cell-derived clones. The sgRNA is indicated above the red line, the PAMs are indicated above the blue line, and the expected cleavage site of SpCas9 is indicated with the black arrow. **e** Log2(FPKM+1) values for all detected genes in Cas9-PKM2 samples versus Cas9-LacZ control. *n* = 3 per group. **f** Schematic of the NLS PKM2 expression cassette and NLS PKM2-GFP fluorescent image. Arrow shows nPKM2 forming a specific punctate-like structure in the nucleus. Experiments were performed three times.


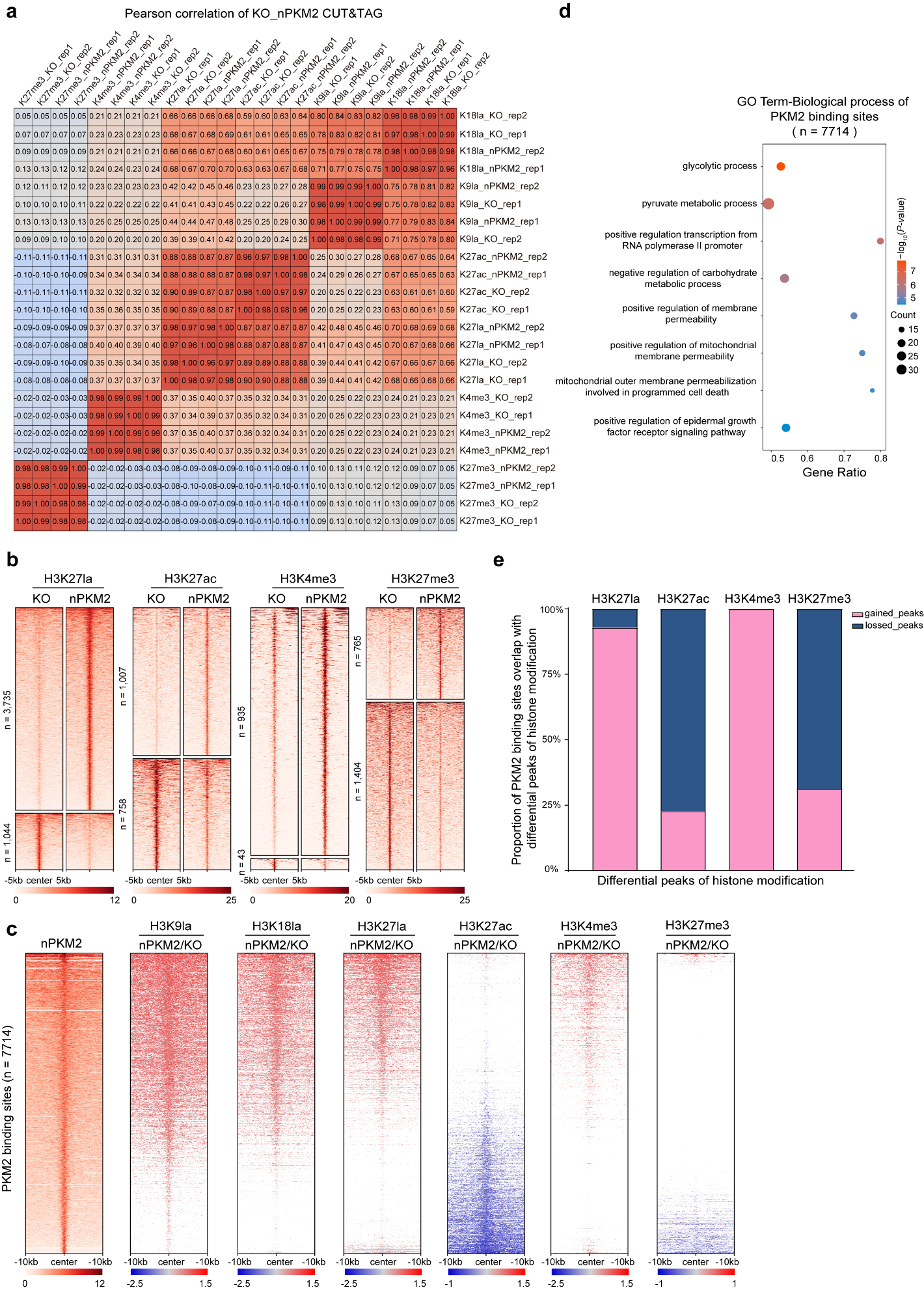


Supplementary Fig. 7

**Nuclear PKM2-induced global histone lactylation in KGN cells.** **a** Correlation heatmap of histone marks for the biological replicates of CUT&TAG experiments, *n* = 2 per group. **b** Heatmap showing differential peak signals of histone lactylation, including H3K27la, H3K27ac, H3K27me3, and H3K4me3 in PKM2 knock out (KO) and nPKM2 cells. The active histone marks were higher in nPKM2 cells (3735 vs. 1044 differential peaks for H3K27la; 1007 vs. 758 for H3K27ac; 765 vs. 1044 for H3K27me3; and 935 vs. 43 for H3K4me3), *n* = 2 per group. **c** Heatmap showing the H3K9la, H3K27ac, H3K27me3, and H3K4me3 signal intensity from +/− 10 kb of PKM2 peak sites in the KO and nPKM2 cells (*n* = 7714), *n* = 2 per group. **d** Gene ontology (GO) biological process enrichment of whole-genomic PKM2-binding sites. **e** Proportion of H3K18la, H3K27la, H3K27ac, H3K4me3, and H3K27me3 differential peaks overlapping with the PKM2-binding sites, *n* = 2 per group.


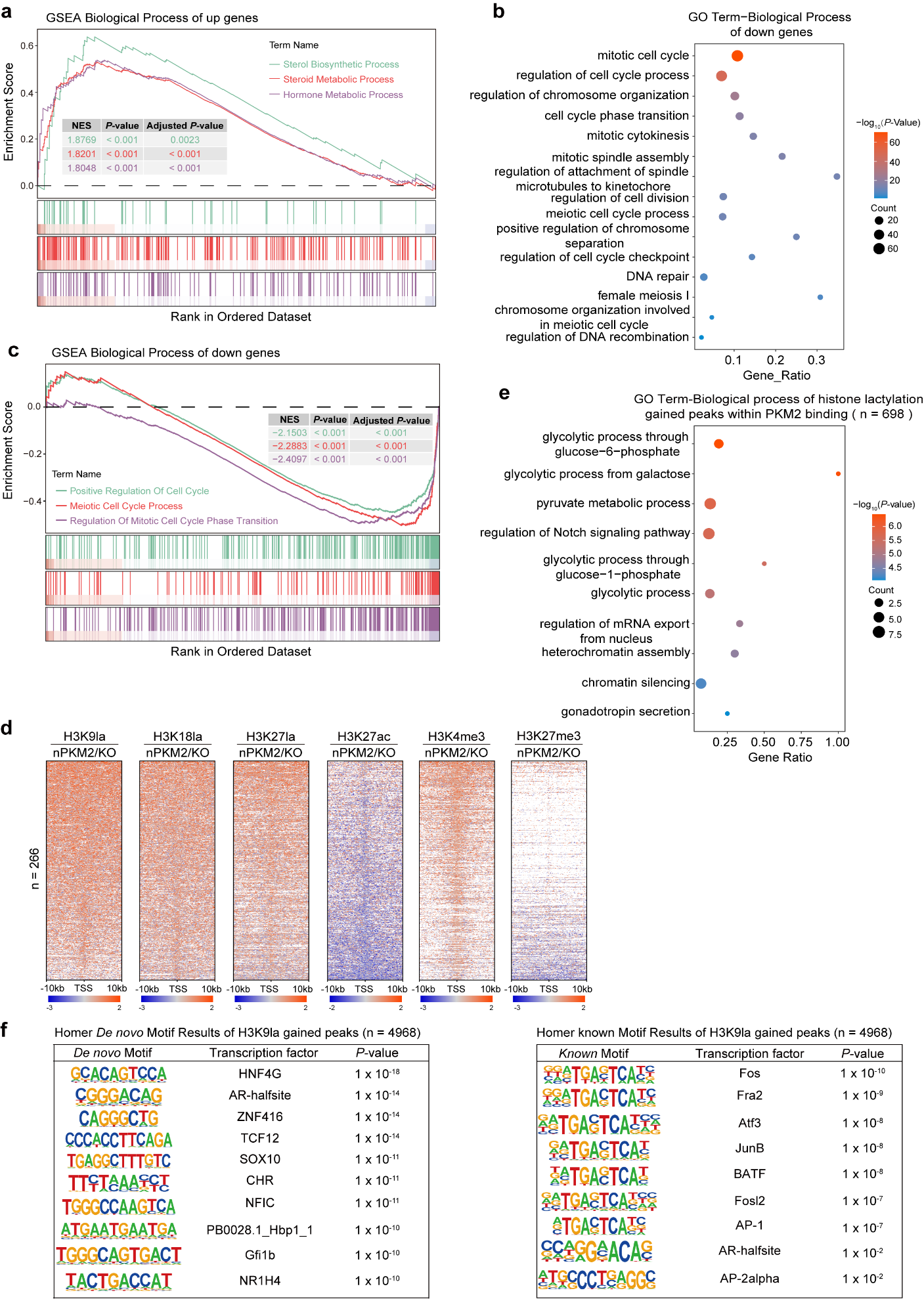


Supplementary Fig. 8

**GSEA analysis of nuclear PKM2 regulated genes and nPKM2-mediated histone lactylation. a** RNA-seq GSEA analysis of upregulated genes in nPKM2 compared with KO cells. Normalized Enrichment Score (NES), False Discovery Rate (*FDR*). Red line shows Steroid Metabolic Process, Green line shows Sterol Biosynthetic Process, Purple line shows Hormone Metabolic Process. **b** Gene ontology (GO) biological process analysis of nPKM2/KO RNA-seq downregulated genes. *n* = 2 in each group. **c** RNA-seq GSEA analysis of downregulated genes in nPKM2 compared with KO cells. Normalized Enrichment Score (NES), False Discovery Rate (*FDR*). Red line shows Meiotic Cell Cycle Process, Green line shows Positive Regulation of Cell Cycle, Purple line shows Regulation of Mitotic Cell Cycle Phase Transition. **d** Heatmap of H3K9la, H3K18la, H3K27la, H3K27ac, H3K4me3, and H3K27me3 signal intensity from +/− 5 kb of nPKM2 upregulated genes transcription start sites (TSS) in KO and nPKM2 cells (*n* = 266). **e** GO biological process enrichment of histone lactylation (K9la and K18la) overlapping with the PKM2-binding sites. *n* = 2 in each group. **f** MOTIF prediction of transcription factor-binding sites in H3K9la gained peaks (*n* = 4968).


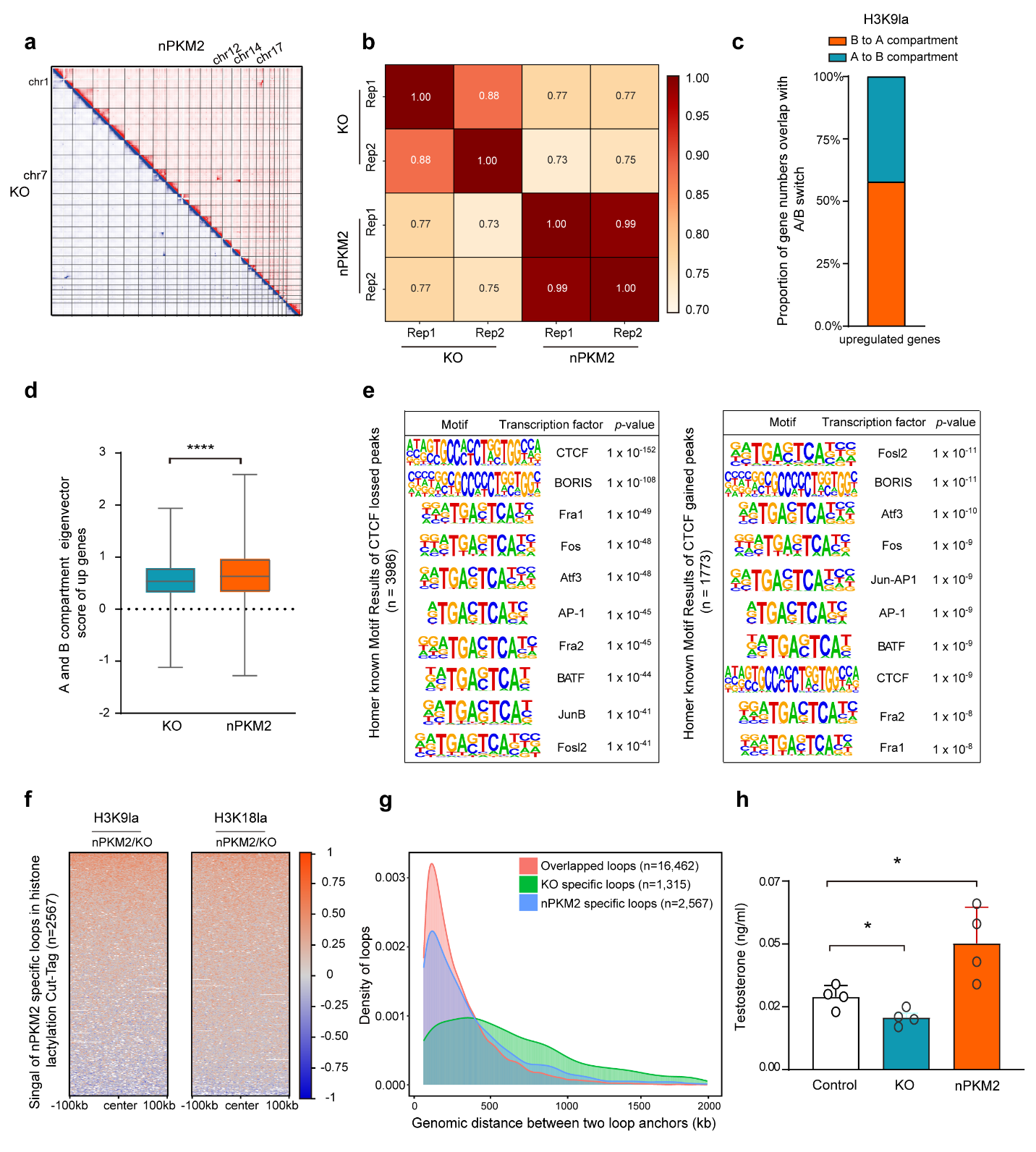


Supplementary Fig. 9

**nPKM2-induced histone lactylation regulates changes in the three-dimensional organization in KO and nPKM2, and determination of testosterone in cells.** **a** Whole-genome heatmap of PKM2 knock out (KO) and nPKM2 Hi-C interaction matrix in KGN cells. **b** Correlation heatmap of Hi-C replicates in PKM2 KO and nPKM2 cells. Biological replicates are highly correlated. *n* = 2 in each group. **c** Proportion of the number of upregulated genes overlapping with A-to-B and B-to-A switched compartments. *n* = 2 in each group. **d** Boxplot showing the global eigenvector score of A and B compartments overlapping with the upregulated genes between KO and nPKM2 cells. The median can be seen in the horizontal line, and the box includes the interquartile range, while the whiskers go up to 5% and 95%. *n* = 2 in each group. **e** Motif analysis of gained and lost peaks of *CTCF*-binding regions in nPKM2 cells. **f** Heatmap of H3K9la and H3K18la signal intensity distribution in nPKM2 cell-specific loop anchors. *n* = 2 in each group. **g** Distance distribution of chromatin loops specific to KO and nPKM2, or those common to both the cell lines. **h** The testosterone concentrations were detected by radioimmunoassay in KO and nPKM2 cells. Experiments were performed three times. Data represented as the mean ± SEM. The *p*-values were determined by Two-tailed unpaired Student’s *t*-test. * *p* < 0.05.


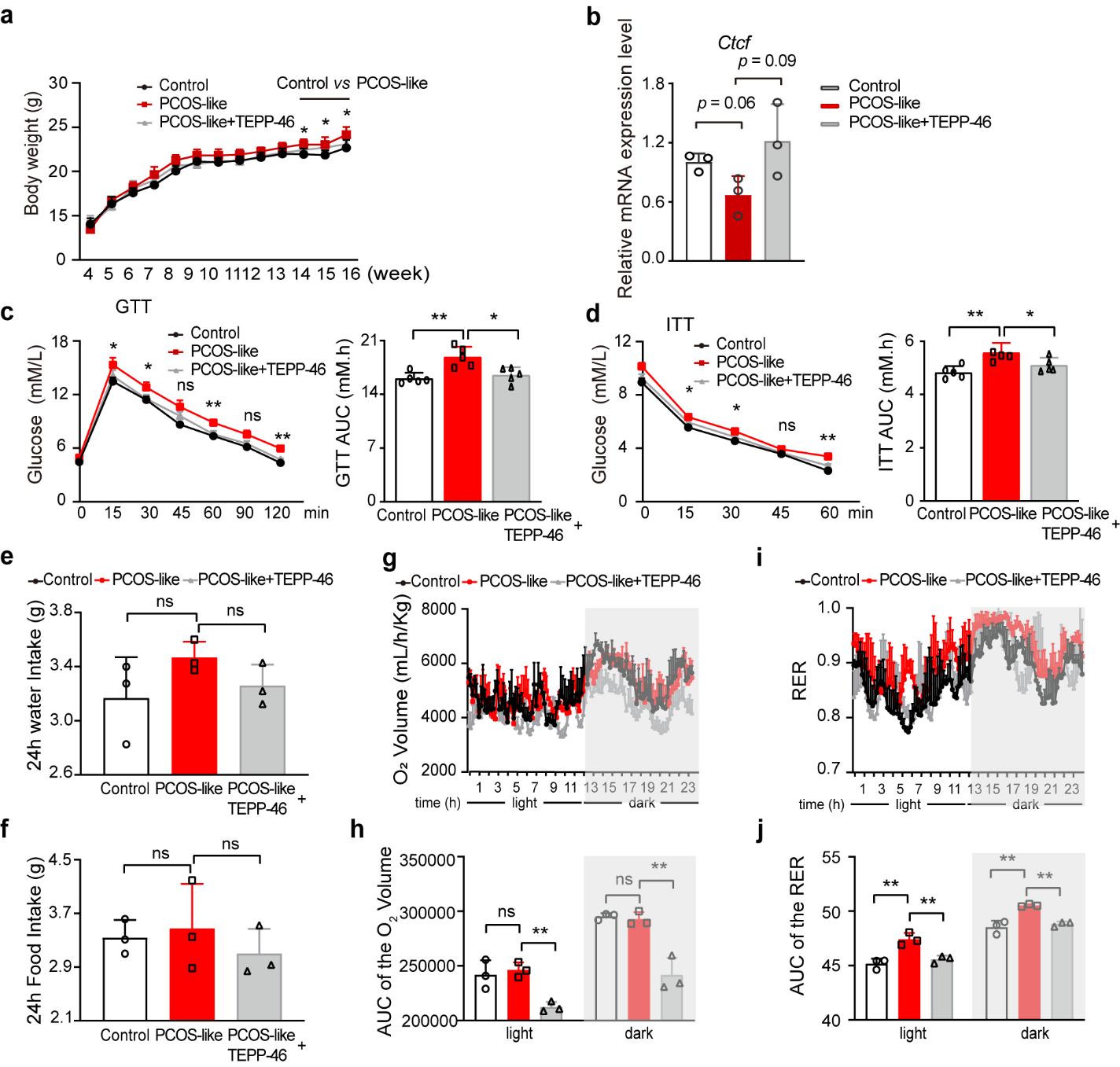


Supplementary Fig. 10

**The TEPP-46 rescues *Ctcf* mRNA expression in the GCs of ovaries and alleviates the metabolic impairment in PCOS-like mice. a** The weight curves for the control, PCOS-like, and PCOS-like+TEPP-46 groups of mice (*n* = 6 per group) were recorded from 4-week to 16-week-old age. Data represented as the mean ± SEM. The *p*-values were determined by Two-tailed unpaired Student’s *t*-test. * *p* < 0.05. **b** The GCs of the ovaries of Control, PCOS-like, and PCOS-like+TEPP-46 mice were checked by qRT-PCR for the level of *Ctcf* mRNA. (*n* = 3 per group). Data represented as the mean ± SEM. The *p* values were determined by a Two-tailed unpaired Student’s *t*-test. **c** Glucose tolerance tests for the Control, PCOS-like and PCOS-like +TEPP-46 mice (*n* = 5 per group). Data represented as the mean ± SEM. The *p* values were determined by a Two-tailed unpaired Student’s *t*-test. ns showed no significant difference. * *p* < 0.05, ** *p* < 0.01. **d** Insulin tolerance test for the three groups (*n* = 5). Data represented as the mean ± SEM. The *p*-values were determined by Two-tailed unpaired Student’s *t*-test. ns showed no significant difference. * *p* < 0.05, ** *p* < 0.01. **e**-**f** The three groups had a normalized body weight after consuming water and food for 24 hours (*n* = 3). Data represented as the mean ± SEM. The *p*-values were determined by a Two-tailed unpaired Student’s *t*-test. ns showed no significant difference. **g**-**h** Estimating the consumed O_2_ in the three groups during both light and dark phases (*n* = 3). Data represented as the mean ± SEM. The *p*-values were determined by Two-tailed unpaired Student’s *t*-test. ns showed no significant difference. ** *p* < 0.01. **i**-**j** Respiratory exchange rates (RER) were detected in the periods of light and darkness for the three groups (*n* = 3). Data represented as the mean ± SD. The *p*-values were determined by a Two-tailed unpaired Student’s *t*-test. ** *p* < 0.01. Experiments were performed three times.

Supplementary Table 1.

The total number of differential proteins in GCs between Control and PCOS.

Supplementary Table 2.

Detection of heterogeneity and directional pleiotropy using MR-Egger regression and Cochran’s *Q* test.

Supplementary Table 3.

The differential expressed genes of RNA-seq data between nPKM2 and KO cells.

Supplementary Table 4.

The ratio of nPKM2 cell-specific loop anchors with or without histone lactylation and overlapped with histone lactylation differential peaks.

Supplementary Table 5.

The TEPP-46 rescued differential genes in the treated mice GCs.

Supplementary Table 6.

Characteristics of the patients in GCs proteomics study.

Supplementary Table 7.

Characteristics of the patients in GCs qPCR study.

Supplementary Table 8.

Characteristics of the patients in GCs RNA-seq study.

Supplementary Table 9.

Characteristics of the patients in GCs plasma UHPLC-MS study.

Supplementary Table 10.

The antibodies, chemicals, Cell lines and Organisms in this study.

Supplementary Table 11.

The primers, sgRNA and other sequences in this study.
