# Supplementary material for: Pyruvate kinase M2-mediated histone lactylation alters three-dimensional genomic architecture in polycystic ovary syndrome
Source: Signal Transduct Target Ther. 2025 Nov 19;10:376. doi: 10.1038/s41392-025-02468-5 (PMC12627565; doi:10.1038/s41392-025-02468-5)

Fig. 1d

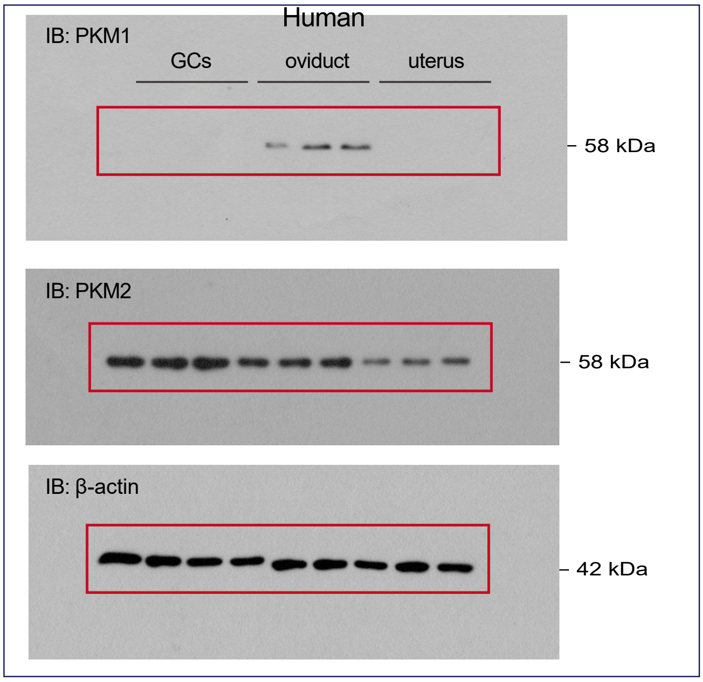

Fig. 2c and d

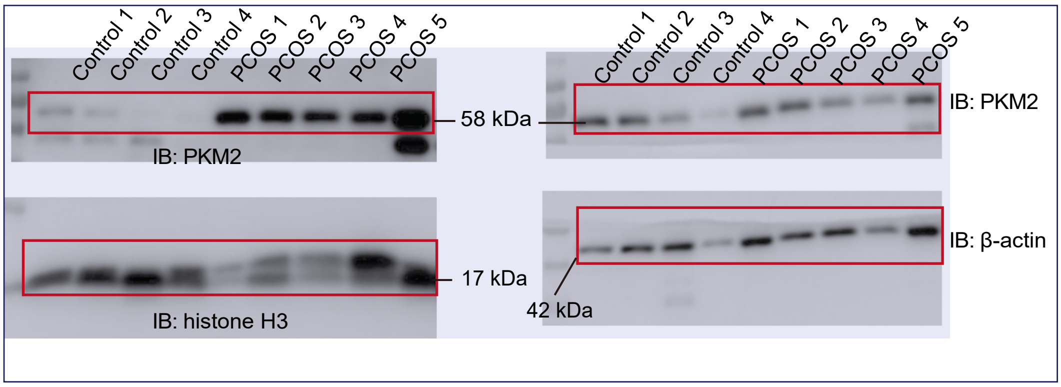

Fig. 2f

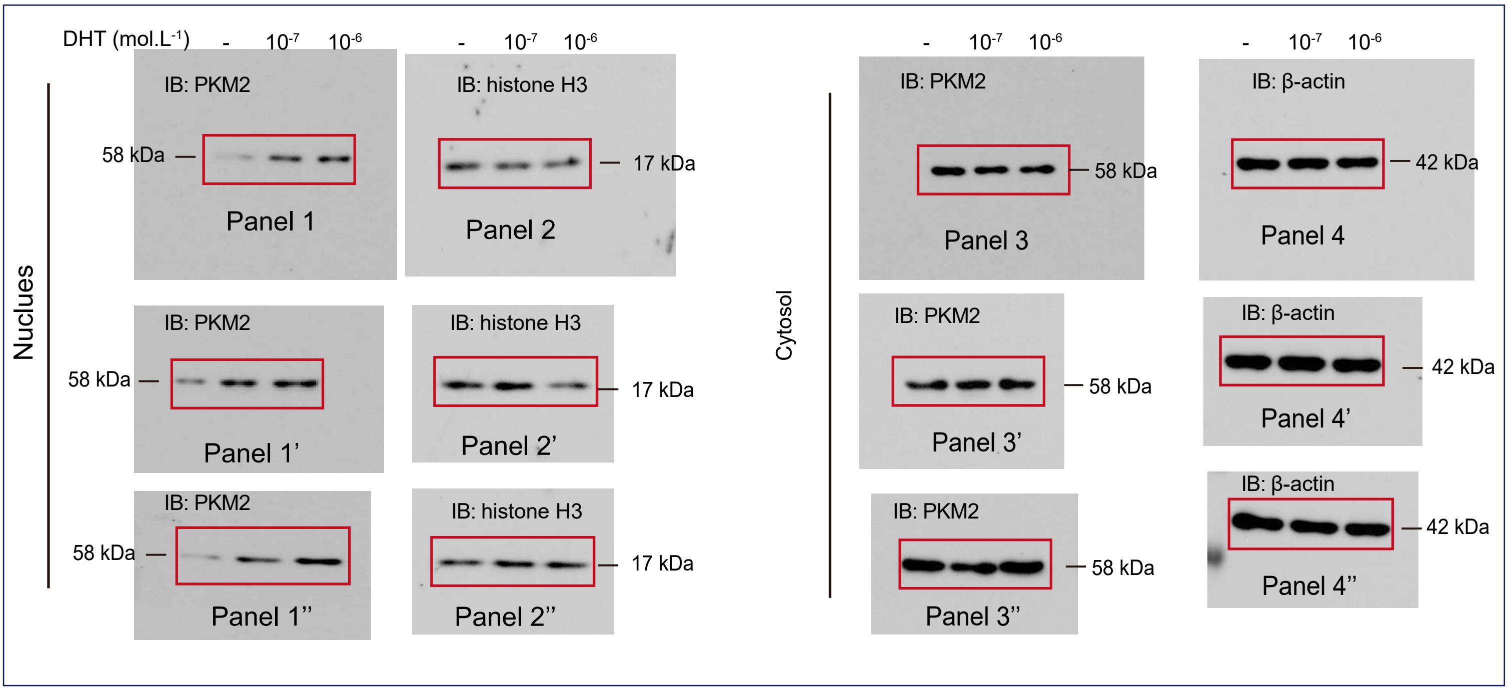

Fig. 2g

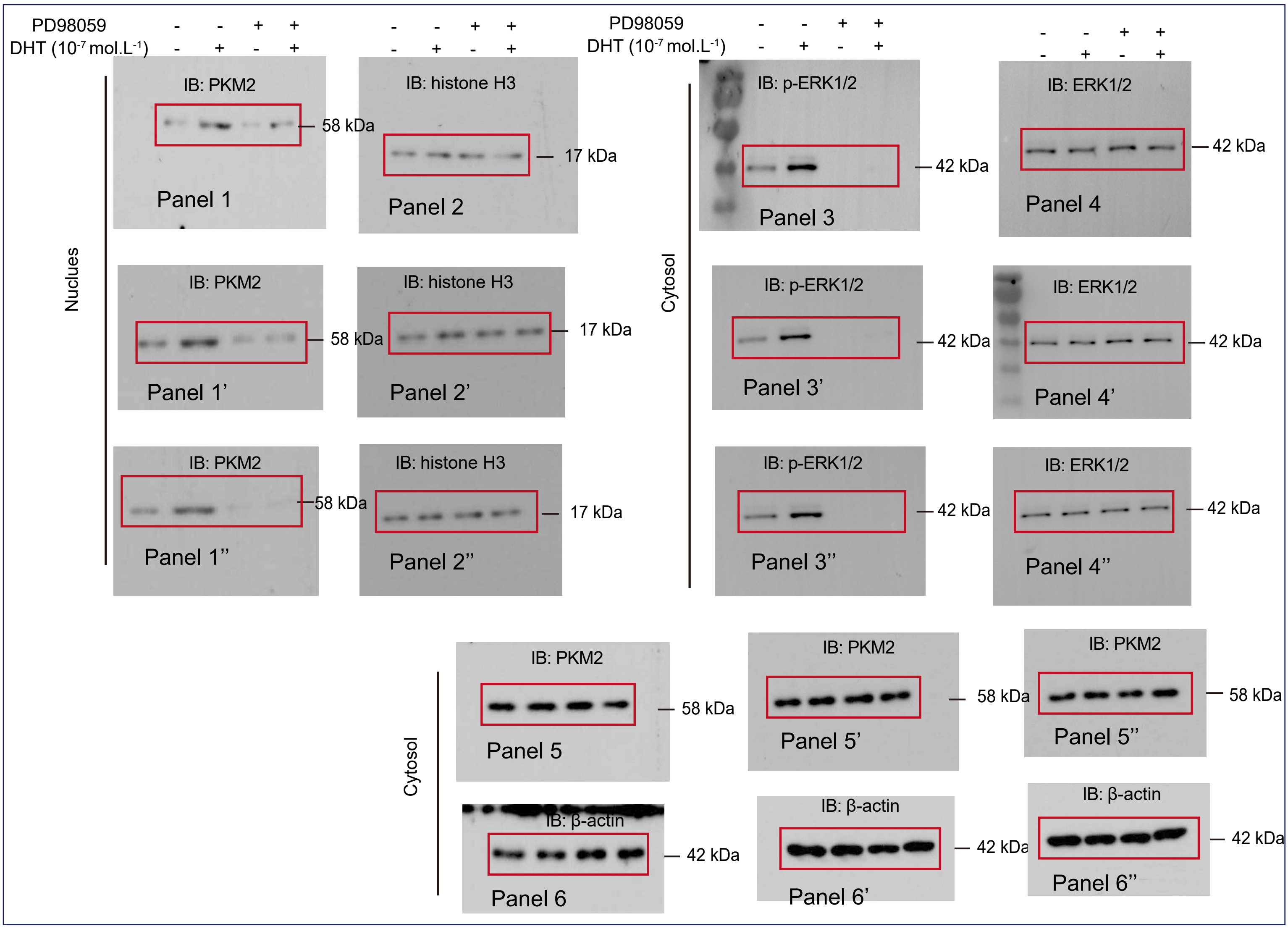

Fig. 4e

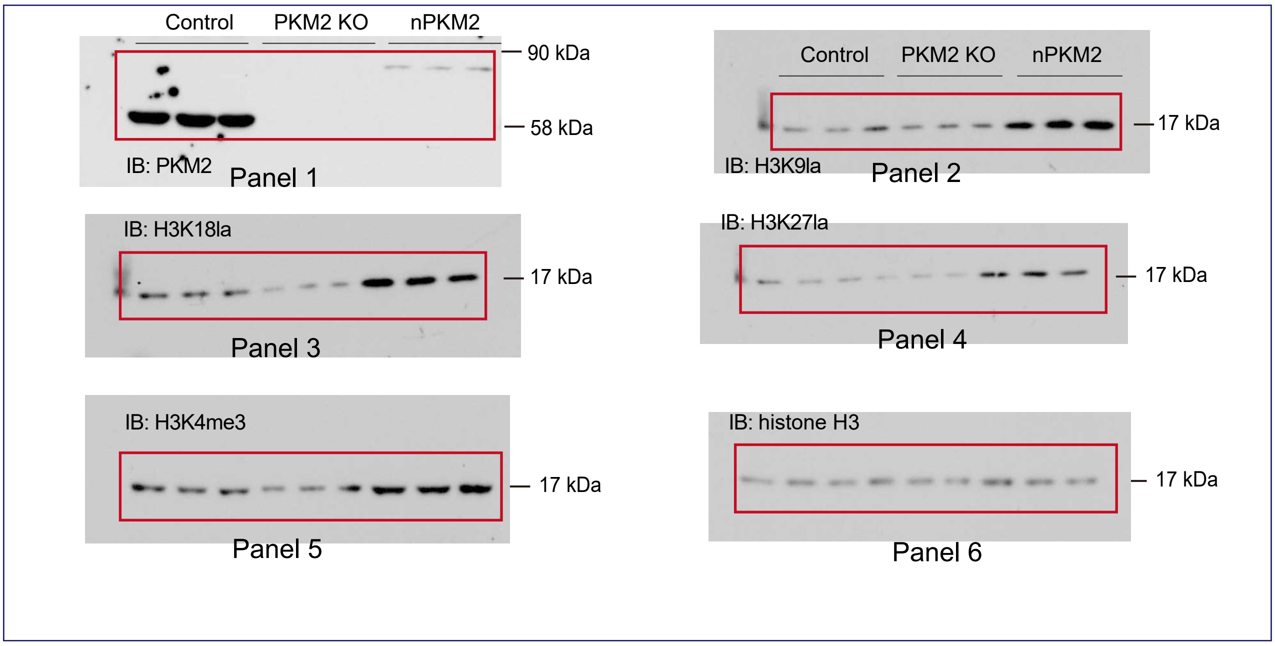

Fig. 4f

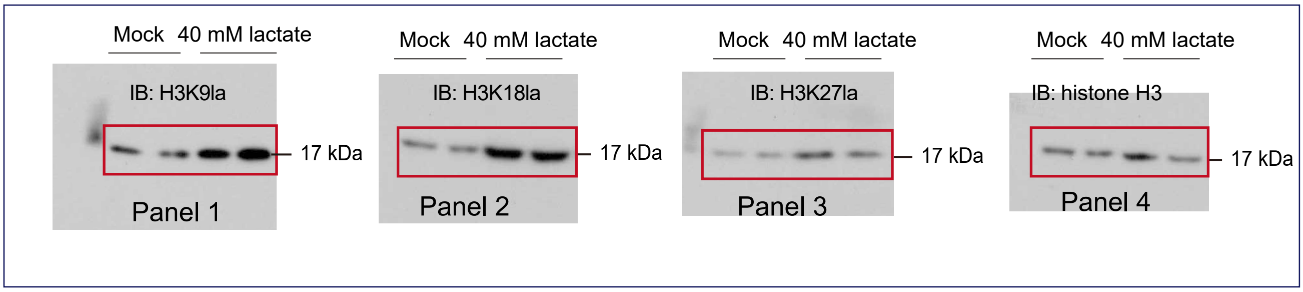

Fig. 6c

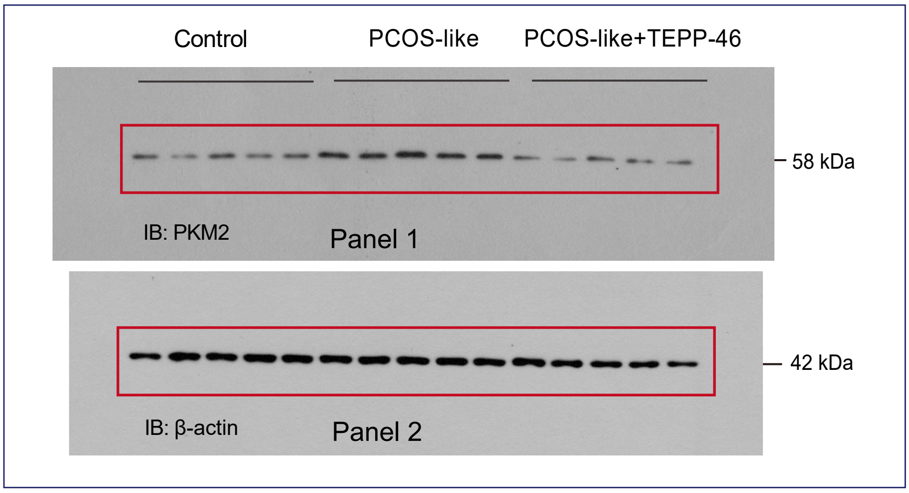

Supplementary Fig. 1b

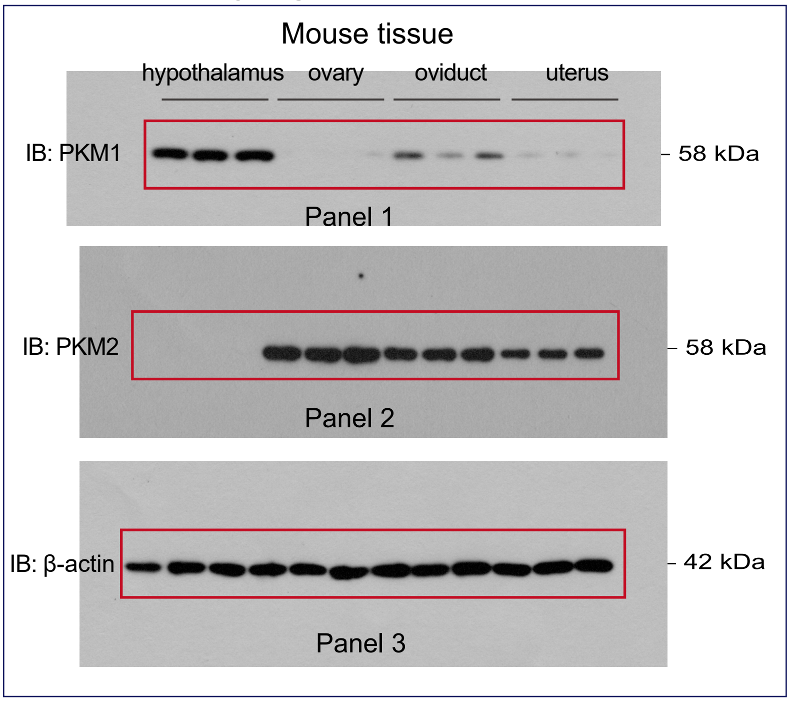

Supplementary Fig. 1c

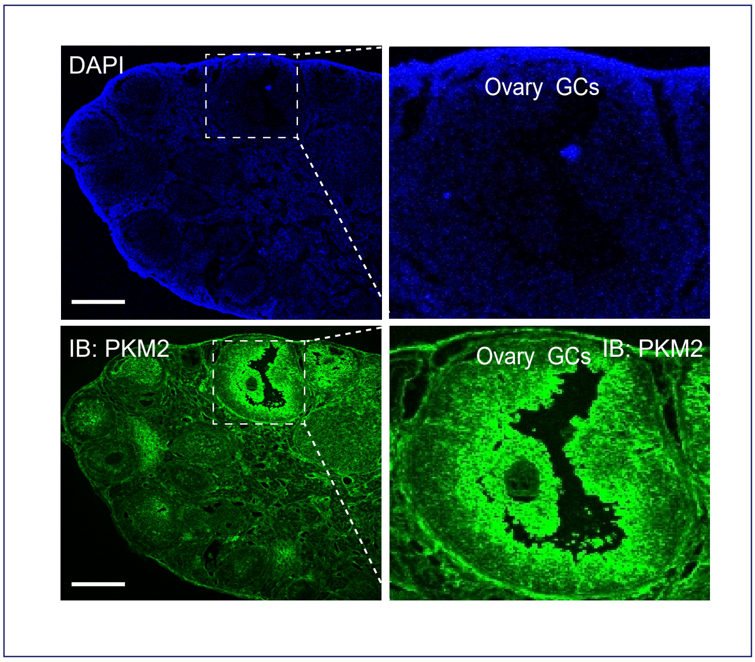

Supplementary Fig. 6c

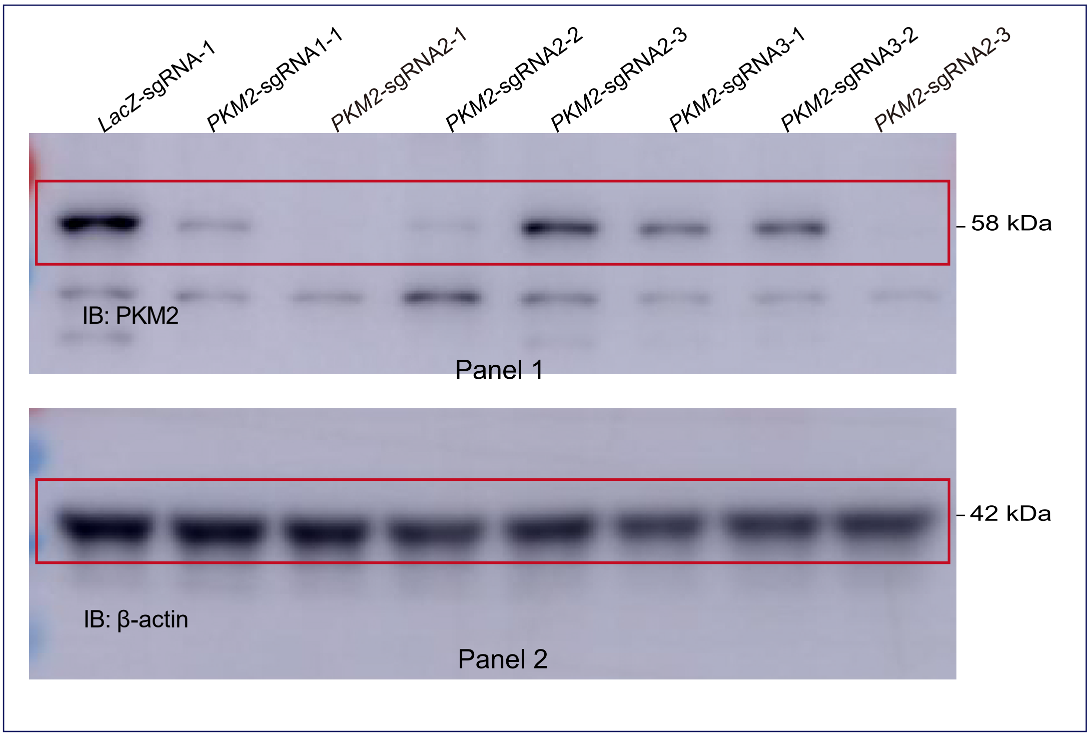

Supplement: Supplementary file 3 — Uncropped films of Western blots [file 41392_2025_2468_MOESM3_ESM.pdf]
